# Adult Children’s Timing of Entry into Parenthood: Parental Investment, Education, and Gender

**DOI:** 10.1007/s12110-025-09502-z

**Published:** 2025-10-25

**Authors:** Vera de Bel, Mirkka Danielsbacka, Markus Jokela, Anna Rotkirch, Antti O. Tanskanen

**Affiliations:** 1 ICS/Department of Sociology, University of Groningen, Groningen, The Netherlands; 2 Netherlands Interdisciplinary Demographic Institute (NIDI), the Hague, the Netherlands; 3 INVEST Research Flagship Centre, University of Turku, Turku, Finland; 4 Population Research Institute (Väestöliitto), Helsinki, Finland; 5 Department of Psychology, University of Helsinki, Helsinki, Finland; 6 Department of Social Research, University of Turku, Turku, Finland

**Keywords:** Parental investment, Intergenerational contact, Timing of entry into parenthood, Age at first birth, Education, Gender

## Abstract

Highly educated individuals have their first child at later ages compared to less-educated individuals, and parental investment is associated with the childbearing of adult children. However, no studies have explored the association between maternal and paternal investment and the timing of parenthood for adult daughters and sons, and whether this association varies by education level. Based on the parenthood penalty and life-history theory, it is hypothesized that parental investment decreases the age at first birth of highly educated adult children and increases the age at first birth of less educated and those currently enrolled in education, particularly between mothers and adult daughters. Event-history analyses were conducted on 4,111 participants and 894 first births from 13 waves of the longitudinal and population-based German Family Panel Analysis of Intimate Relationships and Family Dynamics Study (Pairfam). Results show that contact with mothers was associated with earlier parenthood in less-educated adult children and later parenthood in highly educated adult children. However, contact with fathers was associated with later parenthood in currently enrolled adult children and earlier parenthood in highly educated adult sons. Europe's fertility decline is largely due to delayed age at first birth and parental investment in adult children can contribute to and counteract this trend.

**Supplementary Information:**

The online version contains supplementary material available at 10.1007/s12110-025-09502-z.

## Introduction

Timing of entry into parenthood is an important fertility measure as it affects the number of children parents are able to have overall. In many preindustrial populations, wealthier individuals had children earlier in life compared to their less wealthy counterparts (e.g., Alvergne & Lummaa, 2014). However, in contemporary high-income societies, where education and wealth are strongly associated (Psacharopoulos & Patrinos, 2004), individuals with lower-level education have their first child at a younger age than those with higher-level education do (see Balbo et al., 2013 for an overview). Overall, the age at first birth has increased as infant and child mortality has declined during the demographic transition (e.g., Liu et al., 2012).

The trend of postponing parenthood in contemporary high-income societies applies to all educational groups, but it is most pronounced among the highly educated (Mills et al., 2011; Ní Bhrolcháin & Beaujouan, 2012; Schmidt et al., 2012). Previously, postponement of the first child did not necessarily affect overall cohort fertility, at least not among the highly educated people (Kravdal & Rindfuss, 2008; Sobotka, 2004). However, today, the pattern seems to be changing. In high-income Northern European countries, cohort fertility has declined over the past few decades, and this effect is largely caused by the postponement of first births and a weakening of fertility recuperation (Hellstrand et al., 2021).

Previous studies have indicated that parental investment, defined as any resources or support parents provide to their offspring, including financial, practical, and emotional support (Trivers, 1972), is often, though not always, associated with increased fertility in adult children. These studies have found that parental investment is associated with increased intentions to have children (Tanskanen & Rotkirch, 2014, but see Tanskanen & Danielsbacka, 2021) and actual childbearing (e.g., Aassve et al., 2012; Kaptijn et al., 2010; Schaffnit & Sear, 2017a, b; Tanskanen et al., 2014; Thomese & Liefbroer, 2013; Waynforth, 2012). These studies have also shown that maternal and paternal investment may have different effects on daughters’ and sons’ childbearing.

Only a few studies have investigated whether parental investment is associated with *the timing of entry into parenthood*. Schaffnit and Sear (2017b) used data from the Netherlands and found that reciprocal emotional support between parents and adult children was associated with earlier first births among daughters and, to a lesser extent, among adult sons. Pessin and colleagues (2022), using data from the US, demonstrated that parental time and monetary investment were associated with earlier childbearing among adult daughters, while they did not find the same effect on adult sons. However, in both of these above-mentioned studies, mothers and fathers were grouped into the same category, meaning that their potentially distinct effects could not be differentiated. Moreover, prior studies considering the association between parental investment and fertility rarely considered educational differences in the timing of the first birth.

To the best of our knowledge, this is the first study focusing specifically on how the timing of the first birth depends on the interaction of parental investment and educational group and gender. Therefore, this study investigates the association between parental investment and the age at entry into parenthood for adult children from different educational groups in contemporary Germany. Because providing resources and support requires contact between parents and their adult children, the present study examines intergenerational contact frequency, this can be seen as a proxy or “general” measure for parental investment since it highly correlates with other investment types (e.g., Pollet & Nettle, 2009).

## Theoretical Background

According to inclusive fitness theory (Hamilton, 1964), individuals can increase their own fitness (i.e., number of descendants) by not only reproducing themselves but also supporting the reproductive efforts of close kin. In this sense, parental investment in adult children can be seen as an investment supporting not only the offspring but also the childbearing of their adult children. In accordance with inclusive fitness theory, cooperative breeding theory posits that the human family is a reproductive system where childrearing involves not only the child’s biological parents but also other caretakers, called as allomothers, who often include the adult children’s parents (i.e., the child’s grandparents) (Hrdy, 2009).

Whether parental investment is associated with increased or decreased age at first birth could be highly dependent on adult children’s education level. It is well-documented that the age at entry into parenthood depends on the educational background of an individual, meaning that less-educated individuals often have children at younger ages than their higher-educated counterparts do (e.g., Kravdal & Rindfuss, 2008; Nitsche et al., 2018). In social sciences, differences in the timing of first birth by educational level are often explained by the “parenthood penalty.” This term refers to the asset losses associated with having children, including loss of income and other assets, and typically is more relevant for mothers than fathers (see, for example, Lersch et al., 2017 for a study in the German context). In fact, fathers—compared with men who are childless—may receive a “fatherhood premium” (see Glauber, 2018 for the latest trends).

Because highly educated individuals have invested time and resources in education and may expect to have better-paid jobs than their less-educated counterparts, their financial costs related to earlier childbearing are higher than those of less-educated individuals (Berg et al., 2020), which is consistent with recent findings (England et al., 2016; Glauber, 2018). This may be one reason for postponing the first child, especially for highly educated individuals. If highly educated adult children expect their parents to step in when they enter parenthood—for example, by providing childcare or other forms of investment—this may reduce the parenthood penalty. To receive such investments, however, intergenerational relationships must be active already before a child is born. Therefore, the following hypothesis is proposed:

- H1: Parental investment decreases the age at first birth of highly educated individuals.

The parenthood penalty explains why highly educated individuals tend to postpone parenthood; that is, they postpone wealth losses while their wealth is still increasing. However, evolutionary life-history theory may explain why low-educated individuals tend to advance parenthood. Prior studies using life history theory have considered the trade-offs between current and future childbearing (Coall et al., 2009). For instance, older parents may have more resources and provide a more stable environment for their children than younger ones, but delaying childbearing may lead to decreased fitness, as fertility declines with age, particularly in women. In addition, living in an environment with fewer resources and lower levels of wealth would promote earlier childbearing because it signals a short life expectancy (Nettle, 2011). In low-resource environments, the potential kin helpers also have shorter life expectancy, meaning that postponing childbearing may lead to a decreasing pool of available kin helpers (Berg et al., 2020).

Regarding the association between parental investment and childbearing, studies adopting life history theory have mostly investigated how a father’s absence in early childhood (an indicator of parental investment) is associated with, for instance, the timing of reproduction, lifetime number of children, and age at menarche (Coall et al., 2016). To the best of our knowledge, only one prior study has investigated the association between maternal absence and fertility (Nettle et al., 2011). Although prior studies have mostly examined how (a lack of) early parental investment correlates with future fertility outcomes, the present study analyzes whether the current investment that adult children receive from their parents is associated with childbearing. Theoretically, the impact of early parental investment (or the absence of it) may extend into adulthood because parents who invest heavily during early childhood tend to continue providing support as their children mature (Parrott & Bengtson, 1999; Whitbeck et al., 1991).

Overall, if an individual with lower socioeconomic resources has experienced relatively more parental investment and can expect these investments to continue, this may lead to later reproduction compared to those individuals with lower socioeconomic resources who do not receive as much parental investment. This is because individuals can rely on alloparental help and continue to accumulate their own resources before reproducing. Thus, based on the life-history theory, the following was hypothesized:

- H2: Parental investment increases the age at first birth among individuals who are less educated.

Besides educational differences, gender differences also play a role in the age at first birth. The obligatory costs of reproduction in terms of pregnancy and lactation are always higher for women than for men (Trivers, 1972). Consequently, the availability of help is more crucial for women than for men, and help from a woman’s mother (and other female kin) is often of key importance (Hrdy, 2009). In contemporary Europe, intergenerational relationships are stronger along maternal lines than along paternal lines, and daughters’ mothers are typically the heaviest investors (e.g., de Bel et al., 2025; Perry & Daly, 2017; Tanskanen & Danielsbacka, 2019). Moreover, the parenthood penalty is more pronounced for women, partly because mothers take most of the available parental leave (Cukrowska-Torzewska & Matysiak, 2020; England et al., 2016; Icardi et al., 2022; Liefbroer, 2005). Therefore, based on the different biological and perceived economic costs of reproduction for women and men, the following was hypothesized:

- H3: Parental investment increases the age at first birth more strongly for less-educated adult daughters than for adult sons.
- H4: Parental investment decreases the age at first birth more strongly for highly educated adult daughters than for adult sons.
- H5: The effects of maternal investment are stronger than those of paternal investment.

## Data and Methods

This study analyzed data from Waves 1–13 of the German Panel Analysis of Intimate Relationships and Family Dynamics (pairfam, Brüderl et al., 2021). Pairfam is a longitudinal, multi-actor study that began in 2008 and collects annual data from its respondents. Respondents were drawn from a random national sample covering four birth cohorts: 1971–1973, 1981–1983, 1991–1993, and, since Wave 11, 2001–2003. Although the dataset also contained information on other family members, only data reported by the anchor were used.

### Analytical Sample

Combining the 13 waves of pairfam data initially yielded 99,260 observations from 18,912 respondents. Constructing the analytical sample involved several steps in which some of these observations and respondents were excluded. First, the sample was left-censored, meaning that respondents who already had children at the start of the panel (33,436 observations and 6,136 respondents) were excluded. Second, once respondents had their first child, their following (6,568) observations were also excluded from the sample. Third, a total of 32,430 observations were also excluded. This included (a) respondents who were still living with their parents, (b) cases where it was unknown whether they lived with their parents but who reported living with their parents in the next wave, or (c) the first wave in which they reported living independently. In the latter case, lagged contact (see *Parental investment*) would refer to contact while respondents were still living at home. The 164 births excluded due to this decision belonged to 47 respondents who were less educated, 73 who were medium educated, 23 who were highly educated, and 21 who were currently enrolled.

As this study focuses on the timing of the first birth, longitudinal analyses were performed. Therefore, complete information was needed on the respondents’ birthdate, interview date, birth status, and birthdate of their first child. Accordingly, some observations were additionally excluded because the chronological sequence of events was not logical (178 observations, 52 respondents, and 51 births) or because information on these variables was incomplete (40 observations, 1 respondent, and 35 births). Moreover, for 9,650 observations (2,306 respondents and 360 births), contact was missing “by design”; that is, because this study analyzes lagged contact (see *Parental investment*), contact was missing in the first and second observations for respondents expecting their first child in their first observation. In addition, contact was missing when the parents were deceased. Another 415 observations (98 respondents and 24 births) were excluded due to missing data. All these steps together resulted in an analytical sample of 16,543 observations and 4,111 respondents: 894 respondents had their first child during the panel, and 3,217 respondents did not have a child during the panel. Table 1 presents the descriptive statistics.

### Measurements

#### Age at first birth

This study focused on the age at which the respondents had their first child. First births of biological children were derived from a dichotomization of the variable measuring the number of all biological children born until the time of the interview: no biological children born was coded as 0, and one or more biological children born was coded as 1. For the timing of this event, the study used the birthdate of the oldest biological child if the event had occurred, and the interview date if the event had not yet occurred. The respondents’ birthdate was used as the origin variable, and the date of the first interview served as the entry variable. An additional day was added to the interview date if the time and entry overlapped.

Children’s birthdate information was considered only when it concerned a biological child. If birthdate information was missing or incorrect, the information was supplemented. When the month of birth was missing or incorrect (in 81 and 19 out of 18,912 respondents, respectively), June was imputed as the birth month. The oldest birth date was selected from the list of available birthdates, because the first mentioned biological child was not always the oldest. Ten respondents mentioned a birth date after the interview date; in these cases, the birth date was replaced by the interview date. Table 1 shows that the 1,131 respondents who had their first child during the panel were, on average, 30.97 years old (standard deviation [S.D.]: 4.94).

#### Parental investment

Parental investment is measured by contact frequency between parents and adult children (intergenerational contact), because contact has been shown to indicate overall investment and to correlate with other forms of investment *(e.g.*, Pollet et al., 2009). Contact between adult children and their parents was measured by asking how often the respondents were in contact with both their (biological) mother and father; this included all visits, letters, phone calls, etc. The term “biological” was used in Wave 1, whereas Waves 2–13 mentioned only “mother” or “father,” who could be either a biological or adoptive parent. Because questions about biological parents are not relevant for adopted adult children, the relational reports of respondents who mentioned in Wave 2 that they lived with adoptive parents before the age of six (*N* = 45) or were currently living with an adoptive parent (*N* = 31 for adoptive mothers; *N* = 37 for adoptive fathers) were disregarded in Wave 1.

Respondents could answer (1) *daily*, (2) *several times per week*, (3) *once per week*, (4) *1–3 times per month*, (5) *several times per year*, (6) *less often*, (7) *never*, and (10) *never had contact* to this question. The last category (10) was not included in Wave 1. For the analyses, these contact responses were reverse coded so that 7 represents *daily* contact and 1 represents *never* or *never had contact* (for Waves 2–13). Lagged contact was analyzed by using the score (1–7) reported in the previous wave to predict the age at having the first child. If respondents had their first child in a certain wave and reported in the previous wave that they were already expecting a child (i.e., they or their partner were pregnant), the contact from two waves earlier was used (i.e., the wave before they were expecting a child). This is because contact between the adult child and parent may have already intensified when the child was expected. Table 1 shows that respondents reported more frequent contact with their mothers than with their fathers.

#### Education

Education, a time-varying variable, was measured using the International Standard Classification of Education Index (ISCED, UNESCO, 2006), which was recoded into four groups: First, no degree and lower secondary education were recoded as low education. Second, upper secondary education and post-secondary non-tertiary education were recoded as medium education. Third, the first stage of tertiary education and the second stage of tertiary education were recoded as high education. Fourth, respondents currently enrolled in education were assigned to the fourth group. For this latter group, we cannot determine whether they will ultimately attain a low or high level of education. Nevertheless, we consider it important to include them in the analyses as a distinct category rather than exclude them. Table 1 shows that less-educated respondents are somewhat underrepresented, whereas currently enrolled respondents are slightly overrepresented in the data.

Table 1 also shows the age distribution for different education levels among the 894 respondents who had their first child during the panel. Only 27 currently enrolled respondents had their first child during the panel. Most births occurred in the medium and highly educated groups. The difference in the mean age at first birth between less- and highly educated respondents was almost 10 years.

#### Gender

Differences are expected between less- and highly educated men and women in the timing of having their first child. Therefore, respondents’ gender is an important variable in this study’s model. Since this study also focuses on the potential differences in maternal and paternal contact, it distinguished maternal and paternal contact with their adult daughters and sons. Table 1 shows that the sample consisted of almost equal numbers of men and women.

#### Control variables

The timing of entry into parenthood is likely to be dependent on the presence of a partner. The variable “relationship” distinguishes between respondents who are married, in a civil union, or cohabitating (1), and respondents who are never married, divorced, separated, widowed, or currently not cohabitating (0). Table 1 shows that most respondents do not have a relationship. Because siblings influence each other's fertility (Buyukkececi & Leopold, 2021; Lyngstad & Prskawetz, 2010) and the presence of siblings may reduce the availability of parental investment, the analyses also controlled for whether respondents had siblings. Since visiting divorced parents requires separate visits and because adult children are often less close with divorced fathers later in life (e.g., Kalmijn, 2015; Spaan et al., 2022), it is important to control for the relationship status of the parents. “Parents not together” distinguishes between respondents who ever indicated that their parents were not together, i.e., about one-third of the respondents (see Table 1), and respondents who indicated that their parents were continually together. To check for cultural differences, the models also controlled for whether the respondents were German natives with no history of migration. The variable “DemoDiff” was included to distinguish between respondents who originally stemmed from the DemoDiff subsample, which was collected from East Germany. Lastly, the final set of control variables distinguished between the four birth cohorts. Most respondents were from the 1991–1993 cohort, whereas the youngest (2001–2003) and oldest (1971–1973) cohorts were slightly underrepresented.

**Table 1:** Descriptive statistics (*N* = 16,543 observations, *n* = 4,111 respondents, and 894 Births)

	Mean	S.D.	Min.	Max.	Between S.D.	Within S.D.	N	n
Lagged contact with mother	5.57	1.21	1	7	1.12	0.57	16,543	4,111
Lagged contact with father	4.99	1.54	1	7	.50	0.63	16,543	4,111
	Mean	S.D.	Min.	Max.			N	n
Age at first birth	30.64	4.76	18	47			894	894
Age at first birth by education								
Low	25.52	5.48	18	40			42	42
Medium	29.75	4.46	18	45			422	422
High	32.46	4.08	21	47			403	403
Currently enrolled	25.59	4.27	19	36			27	27
	Proportion		N		n			
Education			16,543		4,111			
Low	3.41		564					
Medium* (reference)*	43.30		7,163					
High	37.96		6,280					
Currently enrolled	15.33		2,536					
Gender (0 = *men*)			16,543		4,111			
Men	50.41		8,339					
Women	49.59		8,204					
Relationship (0 = *no*)			16,543		4,111			
No	46.65		7,717					
Yes	53.35		8,826					
Respondent has siblings (0 = *no*)			16,543		4,111			
No	11.06		1,830					
Yes	88.94		14,713					
German native, no migration (0 = *no*)			16,543		4,111			
No	15.25		2,523					
Yes	84.75		14,020					
Parents ever not together (0 = *no*)			16,543		4,111			
No	65.42		10,823					
Yes	34.58		5,720					
DemoDiff subsample (0 = *no*)			16,543		4,111			
No	91.18		15,084					
Yes	8.82		1,459					
Cohort			16,543		4,111			
1991–1993	37.33		6,176					
1981–1983	46.93		7,764					
1971–1973	15.14		2,505					
2001–2003	0.59		98					

*S.D*. standard deviation; Between S.D. represents differences between individuals in their average scores across measurement times; Within S.D. represents the variability of an individual’s repeated measurements relative to their own average

### Plan of Analyses

Owing to the bell-shaped hazard function, the models were analyzed using a flexible parametric model (Royston & Lambert, 2011). The hypotheses were tested using two- and three-way interactions. Linear combinations of coefficients were calculated to interpret the effects. In additional analyses (Online Supplementary Material (OSM) A), the proportional hazard assumption was tested, after which “relationship” was included as a time-varying component in the model. Contact and gender could not be included as time-varying components, but were accounted for by two- and three-way interactions.

## Results

### Hypotheses Testing

Table 2 presents the results of the flexible parametric model for the age at first birth. Hazard ratios (H.R.s) larger than one indicate that the event of childbirth occurred sooner; that is, respondents were younger. An H.R. below one indicates that the event of childbirth occurred later; that is, respondents were older. An H.R. can also be interpreted as an effect size, because it indicates how much higher or lower the risk of the first birth is for the variable of interest (contact, level of education, gender) compared to its reference at any time during the follow-up time.

The first model, which excluded the control variables, indicated that contact with the father was associated with an H.R. = 1.065 (C.I. = 1.013,1.120) higher probability of childbirth. Such an effect was not found for mothers (H.R. = 1.005; C.I. = 0.942,1.072). Compared to being moderately educated, being currently enrolled in education was associated with a H.R. = 0.355 lower probability (C.I. = 0.237, 0.531) of childbirth, whereas being less educated was associated with a H.R. = 1.578 higher probability (C.I. = 1.148, 2.169) of childbirth. This model also showed that being a woman, compared to being a man, was associated with a H.R. = 1.352 higher probability (C.I. = 1.183,1.545) of childbirth. The direction of these probabilities remained after adding the control variables in Model 2.

Model 3 tested the two-way interactions between contact and education, which enabled testing of the first two hypotheses. Linear combinations of coefficients were used to interpret these interactions (Table 3). The results indicate that maternal contact with less-educated (H.R. = 1.352, C.I. = 1.038, 1.761) and medium-educated (H.R. = 1.126, C.I. = 1.026, 1.236) adult children was associated with earlier childbirth, whereas maternal contact with highly educated (H.R. = 0.805, C.I. = 0.714, 0.909) adult children was associated with later childbirth. These effects are contrary to the expectation that intergenerational contact decreases the age at first birth of highly educated adult children and increases that of less-educated adult children (H1).

The results for fathers were different. Table 3 shows that paternal contact with medium-educated (H.R. = 1.079, C.I. = 1.004, 1.159) and highly educated (H.R. = 1.209, C.I. = 1.086, 1.346) adult children was associated with earlier childbirth. The latter finding is in line with H1, which states that intergenerational contact decreases the age at first birth of highly educated adult children when they are in contact with their fathers. Table 3 shows that paternal contact with currently enrolled adult children (H.R. = 0.767, C.I. = 0.606, 0.970) was associated with later childbirth.

To test the difference between adult daughters and sons, that is, H3 and H4, three-way interactions and all underlying two-way interactions were added to the model. The results (Appendices A and B; Tables 4 and 5, respectively) showed that paternal contact with highly educated adult children was associated with earlier childbirth when these children were sons rather than daughters. It was hypothesized that intergenerational contact decreases the age at first birth more strongly for highly educated daughters than for sons (H4). Therefore, from now onward the findings regarding paternal contact among highly educated adult children can only be interpreted for sons. Moreover, a stronger effect of maternal contact than paternal contact was hypothesized (H5), which was not supported by the results, because contact with fathers, and not mothers, supported the underlying hypotheses H1. Finally, regarding the control variables, being in a relationship and having siblings were associated with earlier childbirth.

### Robustness Checks

The current sample included 894 births, and adding more variables would further limit the sample size due to missing data. Therefore, the possibilities for making further distinctions are limited. Several additional analyses were performed on the model presented in Table 2 to verify the results’ robustness. First, to check whether the results depended on the working status of the adult child and their partner, the study conducted analyses that included a variable indicating whether the anchor or their partner was a housewife or -husband. If the adult child or their partner was a housewife or -husband, the couple would be less dependent on formal and informal childcare, and the hypothesized effect of postponing or advancing childbirth depending on intergenerational relationships may not hold. Including this variable did not change the results (OSM B).

Moreover, two variables indicating whether the adult child’s parents had already reached the age of 66 were added, because the standard retirement age in Germany in 2021 was above 65 years. Previous research in Germany has shown that the retirement status of the parental generation is associated with offspring fertility (Eibich & Siedler, 2020). If future grandparents were still working, they had less time to invest in their offspring, and the hypothesized effect of postponing or advancing childbirth depending on parental investment may not hold. By adding these variables to the model, the association between maternal contact with less-educated adult children and early childbirth disappeared. The association between paternal contact and earlier childbirth of highly educated adult children remained, but the three-way interaction for highly educated adult sons disappeared (OSM C).

Furthermore, on the flip side of availability, future grandparents may also be unavailable to their children and future grandchildren. This may be due to several reasons, such as poor health or vulnerability. In these families, intergenerational contact is expected to be high, but the direction of support is upward rather than downward; that is, the adult child helps the parent. Therefore, additional analyses included two variables in the model indicating whether, during participation in the panel, the respondent had ever indicated that his or her father or mother “needed regular help within the last 12 months.” Including these two variables did not change the results (OSM D).

It was also examined whether the effects differed by cohort. These additional analyses excluded the 2001–2003 cohort because there was only one birth in this cohort. The results showed that the 1981–1983 and 1971–1973 cohorts had children earlier when the respondents had more contact with their fathers. Adult children in the 1991–1993 cohort had children earlier when they had more contact with their mothers (OSM E).

Since contact between parents is strongly correlated when parents are together (Pearson’s correlation = 0.82), the models were re-run using the highest reported intergenerational contact. These models only reproduced the advancement effect of medium-educated adult children (analyses available upon request). Finally, when re-analyzing the models using emotional closeness instead of contact, only the finding that highly educated adult sons’ closeness to fathers was associated with earlier childbirth was replicated (OSM F).

**Table 2:** Flexible parametric model for the age at first birth (*N* = 16,543; Observations, 4, 111 Respondents, and 894 births)

	Model 1		Model 2		Model 3	
	H.R.	C.I.	H.R.	C.I.	H.R.	C.I.
Constant	0.154	[0.106,0.222]	0.019	[0.009,0.040]	0.011	[0.005,0.025]
Lagged maternal contact	1.005	[0.942,1.072]	1.036	[0.967,1.109]	1.126	[1.026,1.236]
Lagged paternal contact	1.065	[1.013,1.120]	1.071	[1.012,1.134]	1.079	[1.004,1.159]
Education						
Low	1.578	[1.148,2.169]	1.794	[1.301,2.475]	1.414	[0.284,7.047]
Medium	*ref.*		*ref.*		*ref.*	
High	0.945	[0.823,1.084]	0.941	[0.819,1.081]	3.432	[1.677,7.024]
Currently enrolled	0.355	[0.237,0.531]	0.470	[0.313,0.706]	0.905	[0.0860,9.525]
Gender (0 = *men*)	1.352	[1.183,1.545]	1.147	[1.003,1.312]	1.165	[1.019,1.333]
*Control variables*						
Relationship (0 = *single*)			8.702	[5.134,14.75]	8.918	[5.300,15.01]
German native, no migration (0 = *no*)			0.958	[0.797,1.152]	0.972	[0.808,1.168]
Parents ever not together (0 = *yes*)			1.089	[0.928,1.277]	1.097	[0.934,1.288]
Respondent has siblings (0 = *yes*)			1.310	[1.046,1.640]	1.319	[1.054,1.652]
DemoDiff subsample (0 = *no*)			1.184	[0.965,1.453]	1.199	[0.977,1.472]
Cohort						
1991–1993			1.058	[0.834,1.343]	1.067	[0.841,1.354]
1981–1983			*ref.*		*ref.*	
1971–1973			0.745	[0.535,1.038]	0.733	[0.526,1.021]
2001–2003			6.305	[0.799,49.73]	6.530	[0.826,51.62]
Education * lag contact interactions						
Lagged maternal contact * low edu					1.200	[0.908,1.588]
Lagged maternal contact * medium edu					*ref.*	
Lagged maternal contact * high edu					0.715	[0.615,0.832]
Lagged maternal contact * enrolled					1.196	[0.790,1.811]
Lagged paternal contact * low edu					0.845	[0.710,1.004]
Lagged paternal contact * medium edu					*ref.*	
Lagged paternal contact * high edu					1.121	[0.988,1.270]
Lagged paternal contact * enrolled					0.711	[0.557,0.906]
Loglikelihood	−413.990		−45.812		−29.072	

Exponentiated coefficients; H.R.: hazard ratio; C.I.: 95% confidence interval; edu: education

**Table 3:** Linear combinations of coefficients: model 3

Education	Coef. main effect lagged contact with mother	Coef. interaction lagged contact with mother * education	Exp. (b)	C.I.
Low	1.126	1.200 =	1.352	[1.038; 1.761]
Medium	1.126	=	1.126	[1.026; 1.236]
High	1.126	0.715 =	0.805	[0.714; 0.909]
Enrolled	1.126	1.196 =	1.347	[0.899; 2.019]
Education	Coef. main effect lagged contact with father	Coef. interaction lagged contact with father * education	Exp. (b)	C.I.
Low	1.079	0.845 =	0.911	[0.776; 1.070]
Medium	1.079	=	1.079	[1.004; 1.159]
High	1.079	1.121 =	1.209	[1.086; 1.346]
Enrolled	1.079	0.711 =	0.767	[0.606; 0.970]

Exponentiated coefficients (coef.); C.I.: 95% confidence interval

## Discussion and Conclusion

This study investigated whether contact between adult children and their parents (i.e., prospective parents and grandparents) is associated with adult children’s timing of entry into parenthood, and whether this varies according to their level of education and gender. Contact frequency was used as a proxy for parental investment. The results showed that parental investment is associated with adult children’s age at entry into parenthood in a high-income society; however, maternal and paternal investment have different implications for the timing of this entry. It was hypothesized that parental investment would be associated with a younger age at childbirth for highly educated adult children and an older age at childbirth for less-educated children. Moreover, stronger effects among women were hypothesized for both generations. The results showed that contact between highly educated adult sons and fathers was indeed associated with a younger age at childbirth. Interestingly, also paternal contact with currently enrolled adult children was associated with delayed childbirth. Contrary to what was hypothesized, contact between highly educated adult children and their mothers was associated with an older age at first birth.

The results of maternal contact contradicted the hypotheses. The results showed that maternal contact with less- and medium-educated adult children was associated with earlier childbirth, and contact with highly educated adult children was associated with later childbirth. These contradictory findings are especially remarkable given that, according to the cooperative breeding literature, the bond between adult daughters and mothers is of key importance for reproductive success. These results could reflect factors not considered in the analyses, such as the mothers’ level of education. For example, highly educated mothers may transfer their values or career attitudes to their highly educated adult children and therefore negatively influence the children’s fertility decisions.

The results for paternal contact partially supported the hypotheses. The results showed that paternal contact with medium-educated adult children and highly educated adult sons was associated with earlier childbirth. Additionally, paternal contact with currently enrolled adult children was associated with later childbirth. For the currently enrolled group, it is important to note that we do not yet know whether they will end up being low, medium, or highly educated. What makes these results especially interesting is that they were not found for maternal contact, whereas the strongest effects were expected in the hypothesized direction between future mothers and their adult daughters. Previous studies have indicated that the father’s absence (i.e., the lack of investment) was associated with teenage pregnancies (Ellis et al., 2003). When considering the potential protective role fathers may play in their children’s lives, it is not surprising that such an effect was found only for future grandfathers; that is, by having contact with their adult children who are still in education, they may help prevent early pregnancy. That said, however, it should also be noted that in present-day affluent societies, father absence is often associated with reproductive factors in girls, but findings regarding boys and premodern or traditional populations are mixed (Sear et al., 2019).

The finding that only contact between highly educated adult sons and their fathers was associated with earlier childbirth is puzzling in two ways. First, why the father? In particular, contact with fathers and mothers was highly correlated when the parents were together. This effect is arguably driven by a positive family climate—that is, by two strong intergenerational relationships. For example, previous research has shown that parental conflict was associated with the postponement of childbearing (Rijken & Liefbroer, 2009). However, this study’s robustness checks analyzing the highest contact only replicated the shared effect of contact with medium-educated children being associated with earlier childbirth. Previous studies have shown that intergenerational relationships differ by gender (de Bel et al., 2025; Fingerman et al., 2020), and the relative importance of paternal involvement may be understudied. When re-analyzing the study’s models by replacing contact with emotional closeness, the same results were found for highly educated adult sons, but not for currently enrolled adult children (OSM F). In addition, this father effect may also be driven by age differences between parents, since in different-sex couples, fathers are usually older, retire earlier, and are available sooner than mothers are. However, the robustness checks did not support this alternative mechanism. This result requires further attention, especially in the normative context of Germany, where fathers are considered the breadwinners. Future research should study whether fathers of highly educated adult children indeed provide more childcare compared to fathers of less-educated adult children and how that affects future fertility behavior (the decision and timing of having another child).

Second, why the son? A stronger effect was hypothesized for adult daughters. A possible explanation for this finding is that the hypothesized parenthood penalty works differently for men and women: women who become mothers pay a motherhood wage penalty (e.g., Budig & England, 2001), whereas men who become fathers earn a fatherhood wage premium (e.g., Hodges & Budig, 2010; Killewald, 2013). Recent research has shown that the motherhood wage penalty decreased, whereas the fatherhood wage premium increased, with the highest premiums observed among highly educated fathers (Glauber, 2018). This premium, along with the role model of strong paternal intergenerational relationships, may explain why highly educated adult sons promote fatherhood when they have greater contact with their fathers.

### Future Research

To disentangle gender differences more precisely, future research should consider adopting a couples’ perspective. This implies that for each (potential) birth, the intergenerational ties of both future parents and their level of education are considered. Although anchor partners are also invited to participate in pairfam, this study did not adopt a couples' perspective because that would also introduce other processes at the couple level that affect fertility, such as patterns of partnering up or downward (Nitsche et al., 2018), which would make the results’ interpretation more complex.

The couples’ perspective also enables the study of a full network of intergenerational relationships. This study considered intergenerational contact with one’s own parents; however, contact with in-laws may affect couples’ fertility to the same extent. Moreover, if one’s own parents do not invest, in-law ties may substitute for the lack of investment with one’s own parents. Future studies adopting a couple’s perspective should consider studying these patterns.

### Limitations

First, this study examined intergenerational contact frequency as a proxy or “general” measure for parental investment, as it highly correlates with other investment types (e.g., Pollet & Nettle, 2009). However, contact may also represent other relational aspects, such as socialization or parental pressure, which could influence the timing of childbirth as parents may shape their adult children’s fertility behaviors. Consequently, there remains uncertainty regarding whether contact genuinely reflects investment. Nonetheless, various robustness checks were conducted that aligned with the reasoning that intergenerational contact suggests some form of parental support for their adult offspring. Second, this study analyzed the first births of respondents in four cohorts:1971–1973, 1981–1983, 1991–1993, and 2001–2003. The latest wave of information was collected in 2020/2021, implying that only the oldest cohort had fully completed their fertile years, whereas only one birth occurred in the youngest cohort. In other words, this study’s analyses are subject to right censoring. If older age groups are completely excluded from the sample, the estimates are likely to be biased and not generalized across the total population. However, because this study also included older cohorts in the analyses, the estimates should not have been affected severely by right censoring. In robustness checks, however, the results differ somewhat for individuals from younger cohorts; that is, the two older cohorts had children earlier when respondents had more contact with their father, whereas the younger cohort (1991–1993) had children earlier when they had more contact with their mother (OSM E). Lastly, the birth status of all siblings was not considered in this study. If adult children’s siblings have already given birth, parents are less likely to invest in their children’s futures. In this analysis, the study controlled for the presence of siblings, which is in fact associated with earlier childbirth.

### Contribution and Implications

The contributions of this study are fourfold: First, to our knowledge, this is the first study to specifically focus on how the timing of the first birth varies by educational group and gender. It investigated the association between paternal and maternal contact and adult sons’ and daughters’ ages at entry into parenthood for different educational groups in contemporary Germany, a high-income society. Second, the study adopted a longitudinal design. Event-history analyses revealed that maternal contact was associated with later childbirth in highly educated adult children, whereas paternal contact was associated with earlier childbirth in highly educated adult sons. Third, the empirical findings contribute to and challenge the parental investment literature. From a cooperative breeding perspective, parental investment contributes to higher reproductive success (Hrdy, 2009). However, this study showed that this contribution to reproductive success depends on individuals’ level of education and gender, since contact between mothers and highly educated adult daughters is associated with later childbirth, and contact between fathers and currently enrolled adult children is associated with later childbirth. In other words, future studies should consider the levels of education and gender before assuming that parental investment indisputably predicts higher reproductive success. Fourth, the present study is linked to a new multidisciplinary research area that combines life history and kin investment theories to examine the effects of family members on different life trajectories (e.g., Coall et al., 2009; Helle et al., 2024).

In summary, this study is the first to investigate the interaction of intergenerational relationships and educational group and gender regarding the timing of first births, a key fertility behavior in all societies, which is also of increasing importance in today’s high-income societies. The decline in European fertility is largely due to later age at first birth. Support from the parents could contribute to and counteract this trend. This study revealed that contact between fathers and their highly educated adult sons may play an important role in lowering their age at first birth.

## Supplementary Information

Below is the link to the electronic supplementary material.

Supplementary Material 1 (PDF 357 KB)

Supplementary Material 2 (PDF 516 KB)

## Data Availability

The data collected by the German Family Panel pairfam (Brüderl et al., 2021) are accessible to the scientific community as scientific use file for scholarly analyses at https://www.pairfam.de/en/data/data-access/.

## Appendix: Model 4 and Three-Way Interactions

**Table 4:** Flexible parametric model for the age at first birth (*N* = 16,543; Observations, 4, 111 Respondents, and 894 births) with Three-Way interactions

	Model 4	
	H.R.	C.I.
Constant	0.020	[0.008,0.050]
Lagged contact with mother	1.010	[0.882,1.157]
Lagged contact with father	1.069	[0.961,1.188]
Education		
Low	0.574	[0.0701,4.703]
Medium	*ref.*	
High	1.912	[0.730,5.008]
Currently enrolled	0.354	[0.0112,11.22]
Gender (0 = *men*)	0.365	[0.133,1.001]
*Control variables*		
Relationship (0 = *single*)	8.880	[5.323,14.82]
German native, no migration (0 = *no*)	0.954	[0.793,1.149]
Parents ever not together (0 = *yes*)	1.103	[0.939,1.296]
Respondent has siblings (0 = *yes*)	1.294	[1.033,1.620]
DemoDiff subsample (0 = *no*)	1.205	[0.981,1.480]
Cohort		
1991–1993	1.043	[0.822,1.325]
1981–1983	*ref.*	
1971–1973	0.731	[0.524,1.018]
2001–2003	6.643	[0.834,52.89]
*Education * lagged contact interactions*		
Lag. cont. with mother * low edu	1.191	[0.795,1.784]
Lag. cont. with mother * medium edu	*ref.*	
Lag. cont. with mother * high edu	0.773	[0.621,0.962]
Lag. cont. with mother * enrolled	1.160	[0.579,2.325]
Lag. cont. with father * low edu	1.034	[0.784,1.364]
Lag. cont. with father * medium edu	*ref.*	
Lag. cont. with father * high edu	1.190	[0.987,1.435]
Lag. cont. with father * enrolled	0.895	[0.550,1.454]
*Education * gender interactions*		
Women * low edu	9.857	[0.360,269.8]
Women * medium edu	*ref.*	
Women * high edu	2.899	[0.676,12.43]
Women * enrolled	6.817	[0.0608,764.5]
*Gender * lagged contact interactions*		
Lag. cont. with mother * women	1.215	[1.005,1.469]
Lag. cont. with father * women	1.034	[0.900,1.188]
*Three-way interactions*		
Lag. cont. with mother * daughter * low edu	0.993	[0.557,1.773]
Lag. cont. with mother * daughter * medium edu	*ref.*	
Lag. cont. with mother * daughter * high edu	0.901	[0.664,1.224]
Lag. cont. with mother * daughter * enrolled	0.961	[0.399,2.318]
Lag. cont. with father * daughter * low edu	0.636	[0.442,0.916]
Lag. cont. with father * daughter * medium edu	*ref.*	
Lag. cont. with father * daughter * high edu	0.866	[0.673,1.114]
Lag. cont. with father * daughter * enrolled	0.708	[0.402,1.245]
Log likelihood	−18.810	

Exponentiated coefficients; *H.R*. hazard ratio; *C.I. *95% confidence interval; *Lag. cont.* lagged contact; *edu* education

## Appendix B: Linear Combinations Model 4

**Table 5:** Linear combinations of coefficients: model 4

Lagged contact with father by education and gender of the adult child	Exp. (b)	C.I.	
*Adult sons*			
Low	1.105	[0.854; 1.430]	
Medium	1.069	[0.961; 1.188]	
High	1.272	[1.087; 1.487]	
Enrolled	0.956	[0.594; 1.538]	
*Adult daughters*			
Low	0.404	[0.143; 1.142]	
Medium	0.390	[0.140; 1.089]	
High	0.465	[0.168; 1.288]	
Enrolled	0.349	[0.115; 1.064]	

Exponentiated coefficients (coef.); C.I.: 95% confidence interval
