# Supplementary material for: Adult Children’s Timing of Entry into Parenthood: Parental Investment, Education, and Gender
Source: Hum Nat. 2025 Oct 25;36(3):482–502. doi: 10.1007/s12110-025-09502-z (PMC12644179; doi:10.1007/s12110-025-09502-z)
Supplement: Supplementary file 2 — Supplementary Material 2 (PDF 516 KB) [file 12110_2025_9502_MOESM2_ESM.pdf]

Online Supplementary Materials

de Bel et al. (2025) Adult Children's Timing of Entry into Parenthood: Parental Investment, Education, and Gender. ***Human Nature***

Online Supplementary Material A 2
Model 3 (presented in Table 2) 2
Model 4 (presented in Table 4) 4
Online Supplementary Material B 8
Model 3 (presented in Table 2) 8
Linear combinations (presented in Table 3) 9
Model 4 (presented in Table 4) 11
Linear combinations (presented in Table 5) 14
Online Supplementary Material C 17
Model 3 (presented in Table 2) 17
Linear combinations (presented in Table 3) 19
Model 4 (presented in Table 4) 21
Linear combinations (presented in Table 5) 23
Online Supplementary Material D 26
Model 3 (presented in Table 2) 26
Linear combinations (presented in Table 3) 27
Model 4 (presented in Table 4) 29
Linear combinations (presented in Table 5) 32
Online Supplementary Material E 34
Model 35
Linear combinations 36
Online Supplementary Material F 39
Model 3 (presented in Table 2) 39
Linear combinations (presented in Table 3) 40
Model 4 (presented in Table 4) 42
Linear combinations (presented in Table 5) 45

Online Supplementary Material A

Testing the proportional hazard assumption

Model 3 (presented in Table 2)

```
. stcox c.contactm_lag##b2.isced_rec c.contactf_lag##b2.isced_rec sex rela german_native_nomigration
parents_evernottogether siblings demodiff b2.cohort, nolog


         Failure _d: have_kids==1
   Analysis time _t: (time-origin)/365
             Origin: time birthdate_respondent
  Enter on or after: time first_obs
  Exit on or before: time .
        ID variable: id


Cox regression with Breslow method for ties


No. of subjects =       4,111                         Number of obs = 16,543
No. of failures =         894
Time at risk    = 25,328.8658
                                                      LR chi2(20)   = 828.82
Log likelihood = -5658.6964                           Prob > chi2   = 0.0000


----------------------------------------------------------------------------------------
                      _t | Haz. ratio   Std. err.      z    P>|z|     [95% conf. interval]
-------------------------+--------------------------------------------------------------
             contactm_lag |   1.122407   .0533531     2.43   0.015      1.02256    1.232003
                         |
               isced_rec |
                       1  |   1.294118   1.061231     0.31   0.753     .2593895    6.456468
                       3  |   3.338325    1.21745     3.31   0.001     1.633446     6.82264
                       4  |   .8994174   1.081217    -0.09   0.930     .0852524    9.488908
                         |
 isced_rec#c.contactm_lag |
                       1  |   1.214674    .173502     1.36   0.173     .9180692    1.607103
                       3  |   .7221502   .0552126    -4.26   0.000     .6216532    .8388935
                       4  |   1.206327   .2547673     0.89   0.374     .7974396    1.824873
                         |
             contactf_lag |   1.076856   .0397464     2.01   0.045     1.001705    1.157644
```

```
                             |
  isced_rec#c.contactf_lag |
                         1  |   .8453382   .0742684    -1.91   0.056     .7116181    1.004186
                         3  |   1.115098    .070933     1.71   0.087     .9843896    1.263163
                         4  |   .7099977   .0886416    -2.74   0.006     .5558868    .9068335
                             |
                         sex |   1.166055   .0800207     2.24   0.025     1.019308     1.33393
                        rela |   15.90798   2.496792    17.63   0.000     11.69546     21.6378
 german_native_nomigration |   .9719589    .091453    -0.30   0.762     .8082714    1.168796
   parents_evernottogether |    1.08557   .0890394     1.00   0.317     .9243608    1.274894
                    siblings |   1.322864   .1517567     2.44   0.015     1.056494    1.656394
                    demodiff |    1.18995    .124619     1.66   0.097     .9691381    1.461073
                             |
                      cohort |
               1 1991-1993  |   1.092675   .1345588     0.72   0.472     .8583584    1.390955
               3 1971-1973  |   .5892256   .1077765    -2.89   0.004     .4117053    .8432896
               4 2001-2003  |    9.05678   9.621011     2.07   0.038     1.129128    72.64481
-------------------------------------------------------------------------------------------

. estat phtest, detail

Test of proportional-hazards assumption

Time function: Analysis time
--------------------------------------------------------
             |       rho     chi2       df    Prob>chi2
-------------+------------------------------------------
contactm_lag |  -0.03249     1.00        1       0.3165
 1.isced_rec |  -0.00883     0.08        1       0.7818
2b.isced_rec |         .        .        1            .
 3.isced_rec |   0.02874     0.74        1       0.3893
 4.isced_rec |   0.04506     1.29        1       0.2559
1.isced_re~g |   0.01271     0.15        1       0.6981
2b.isced_r~g |         .        .        1            .
3.isced_re~g |   0.05071     2.55        1       0.1102
4.isced_re~g |  -0.03005     0.57        1       0.4520
contactf_lag |   0.05610     2.98        1       0.0841
1.isced_re~g |  -0.06835     4.01        1       0.0452
2b.isced_r~g |         .        .        1            .
```

```
3.isced_re~g |   -0.04654     2.15        1       0.1428
4.isced_re~g |   -0.00896     0.06        1       0.8005
         sex |   -0.15908    22.29        1       0.0000
        rela |    0.10950    10.63        1       0.0011
german_nat~n |    0.08527     6.58        1       0.0103
parents_ev~r |   -0.01912     0.32        1       0.5715
    siblings |   -0.01983     0.35        1       0.5526
    demodiff |   -0.05450     2.66        1       0.1026
    1.cohort |   -0.03191     0.86        1       0.3536
   2b.cohort |          .        .        1            .
    3.cohort |    0.00754     0.05        1       0.8247
    4.cohort |   -0.00135     0.00        1       0.9682
-------------+---------------------------------------
 Global test |              145.53       20       0.0000
--------------------------------------------------------
```

Model 4 (presented in Table 4)

```
. stcox c.contactm_lag##b2.isced_rec##sex c.contactf_lag##b2.isced_rec##sex rela
german_native_nomigration parents_evernottogether siblings demodiff b2.cohort, nolog


         Failure _d: have_kids==1
   Analysis time _t: (time-origin)/365
             Origin: time birthdate_respondent
  Enter on or after: time first_obs
  Exit on or before: time .
        ID variable: id


Cox regression with Breslow method for ties


No. of subjects =       4,111                         Number of obs = 16,543
No. of failures =         894
Time at risk    = 25,328.8658
                                                      LR chi2(31)   = 848.65
Log likelihood = -5648.7789                           Prob > chi2   = 0.0000


---------------------------------------------------------------------------------------------
                          _t | Haz. ratio   Std. err.      z    P>|z|     [95% conf. interval]
----------------------------+----------------------------------------------------------------
                contactm_lag |      1.004   .0695273     0.06   0.954     .8765724    1.149952
```

isced_rec						
1	.5439166	.5777562	-0.57	0.566	.0678225	4.362054
3	1.809282	.8884947	1.21	0.227	.6910376	4.737081
4	.3150791	.5598598	-0.65	0.516	.0096812	10.25435
isced_rec#c.contactm_lag						
1	1.210179	.2481626	0.93	0.352	.8096547	1.808836
3	.7906749	.0874762	-2.12	0.034	.6365396	.9821333
4	1.19491	.4281674	0.50	0.619	.5920082	2.411809
1.sex	.3646602	.1874248	-1.96	0.050	.1331666	.9985767
sex#c.contactm_lag						
1	1.220348	.1184582	2.05	0.040	1.008924	1.476077
isced_rec#sex						
1 1	9.260442	15.75931	1.31	0.191	.3296604	260.1337
3 1	3.045919	2.257926	1.50	0.133	.7124041	13.02297
4 1	8.133037	19.71792	0.86	0.387	.0702393	941.7277
isced_rec#sex#c.contactm_lag						
1 1	.9828093	.2921139	-0.06	0.953	.5488735	1.759812
3 1	.8796972	.1364883	-0.83	0.409	.6490319	1.192341
4 1	.9279646	.4201413	-0.17	0.869	.3820718	2.253812
contactf_lag	1.069301	.0580051	1.24	0.217	.9614477	1.189252
isced_rec#c.contactf_lag						
1	1.026135	.143382	0.18	0.854	.7803075	1.349407
3	1.17358	.111064	1.69	0.091	.9748946	1.412758
4	.8956185	.2254967	-0.44	0.661	.5467762	1.467022
sex#c.contactf_lag						
1	1.030011	.0731925	0.42	0.677	.8960981	1.183936
isced_rec#sex#c.contactf_lag						
1 1	.647877	.1199788	-2.34	0.019	.450672	.9313749
3 1	.8802019	.1122401	-1.00	0.317	.6855518	1.13012

```
                      4 1  |   .7070709   .2062671    -1.19   0.235     .3991627    1.252495
                           |
                      rela |   15.87707   2.495018    17.59   0.000      11.6683    21.60396
  german_native_nomigration |   .9559073   .0902981    -0.48   0.633     .7943438    1.150332
    parents_evernottogether |   1.092914   .0899671     1.08   0.280     .9300719    1.284268
                   siblings |   1.297138    .148983     2.27   0.024      1.03567    1.624617
                   demodiff |   1.197268   .1257177     1.71   0.086     .9745681    1.470859
                           |
                     cohort |
               1 1991-1993  |   1.071666   .1322949     0.56   0.575     .8413575    1.365019
               3 1971-1973  |   .5875362   .1074426    -2.91   0.004     .4105591    .8408017
               4 2001-2003  |   9.436867   10.03549     2.11   0.035     1.173899    75.86209
----------------------------------------------------------------------------------------------

. estat phtest, detail

Test of proportional-hazards assumption

Time function: Analysis time
--------------------------------------------------------
             |       rho     chi2       df    Prob>chi2
-------------+------------------------------------------
contactm_lag |    0.01171     0.14        1       0.7076
 1.isced_rec |    0.01121     0.13        1       0.7156
2b.isced_rec |          .        .        1            .
 3.isced_rec |    0.03701     1.29        1       0.2564
 4.isced_rec |    0.01535     0.11        1       0.7348
1.isced_re~g |    0.00120     0.00        1       0.9648
2b.isced_r~g |          .        .        1            .
3.isced_re~g |   -0.00949     0.09        1       0.7673
4.isced_re~g |   -0.02507     0.27        1       0.6046
      0b.sex |          .        .        1            .
       1.sex |   -0.01245     0.14        1       0.7099
0b.sex#co.~g |          .        .        1            .
1.sex#c.co~g |   -0.03487     1.14        1       0.2853
1o.isced_r~x |          .        .        1            .
1.isced_re~x |   -0.01660     0.28        1       0.5981
2b.isced_r~x |          .        .        1            .
2b.isced_r~x |          .        .        1            .
```

```
3o.isced_r~x |          .        .         1           .
3.isced_re~x |   -0.00855      0.06        1       0.7999
4o.isced_r~x |          .        .         1           .
4.isced_re~x |    0.03545      0.73        1       0.3924
1o.isced_r~g |          .        .         1           .
1.isced_re~g |    0.02447      0.60        1       0.4401
2b.isced_r~g |          .        .         1           .
2b.isced_r~g |          .        .         1           .
3o.isced_r~g |          .        .         1           .
3.isced_re~g |    0.04989      2.39        1       0.1219
4o.isced_r~g |          .        .         1           .
4.isced_re~g |   -0.01023      0.05        1       0.8162
contactf_lag |    0.01384      0.18        1       0.6720
1.isced_re~g |   -0.04613      2.06        1       0.1509
2b.isced_r~g |          .        .         1           .
3.isced_re~g |    0.00026      0.00        1       0.9933
4.isced_re~g |    0.01199      0.10        1       0.7483
0b.sex#co.~g |          .        .         1           .
1.sex#c.co~g |    0.03941      1.49        1       0.2217
1o.isced_r~g |          .        .         1           .
1.isced_re~g |   -0.03138      0.95        1       0.3289
2b.isced_r~g |          .        .         1           .
2b.isced_r~g |          .        .         1           .
3o.isced_r~g |          .        .         1           .
3.isced_re~g |   -0.04750      2.23        1       0.1351
4o.isced_r~g |          .        .         1           .
4.isced_re~g |   -0.02205      0.36        1       0.5511
        rela |    0.11168     11.06        1       0.0009
german_nat~n |    0.08638      6.80        1       0.0091
parents_ev~r |   -0.01960      0.34        1       0.5614
    siblings |   -0.02085      0.39        1       0.5314
    demodiff |   -0.05258      2.48        1       0.1156
    1.cohort |   -0.03020      0.78        1       0.3782
   2b.cohort |          .        .         1           .
    3.cohort |    0.00774      0.05        1       0.8203
    4.cohort |    0.00086      0.00        1       0.9797
-------------+---------------------------------------------
 Global test |               143.96       31       0.0000
-----------------------------------------------------------
```

Online Supplementary Material B

Include housewife/househusband

Model 3 (presented in Table 2)

```
. eststo isced3: stpm2 c.contactm_lag##b2.isced_rec c.contactf_lag##b2.isced_rec sex rela
german_native_nomigration parents_evernottogether siblings demodiff b2.cohort house, scale(hazard)

> df(5) tvc(rela) dftvc(5) eform

note: delayed entry models are being fitted


Iteration 0:   log likelihood = -42.859897

Iteration 1:   log likelihood = -31.250432

Iteration 2:   log likelihood = -23.267345

Iteration 3:   log likelihood = -21.707017

Iteration 4:   log likelihood =  -21.69628

Iteration 5:   log likelihood = -21.696277

Iteration 6:   log likelihood = -21.696277


Log likelihood = -21.696277                             Number of obs = 16,533


------------------------------------------------------------------------------------------

                          |     exp(b)   Std. err.      z    P>|z|     [95% conf. interval]

--------------------------+---------------------------------------------------------------

xb                        |

             contactm_lag |   1.132141   .0539378     2.61   0.009      1.03121     1.24295

                          |

                isced_rec |

                       1  |   1.359902   1.114143     0.38   0.707     .2729818    6.774568

                       3  |   3.618031   1.324826     3.51   0.000     1.765164    7.415824

                       4  |    .942152    1.13388    -0.05   0.961     .0890636    9.966478

                          |

 isced_rec#c.contactm_lag |

                       1  |    1.17109   .1674815     1.10   0.269     .8848244     1.54997

                       2  |          1   3.97e-16    -0.04   0.972            1           1

                       3  |   .7120095   .0548774    -4.41   0.000     .6121816    .8281161

                       4  |   1.195417   .2535628     0.84   0.400     .7888042    1.811632

                          |

             contactf_lag |   1.079801   .0399228     2.08   0.038     1.004321    1.160953

                          |

 isced_rec#c.contactf_lag |
```

```
                          1  |   .8674104   .0790508    -1.56   0.119     .7255227    1.037047
                          3  |   1.116616   .0714907     1.72   0.085      .984932    1.265906
                          4  |   .7093267   .0881784    -2.76   0.006      .555944    .9050271
                             |
                         sex |   1.165724   .0799975     2.23   0.025     1.019019     1.33355
                        rela |    9.66023   2.365116     9.26   0.000     5.978436    15.60944
german_native_nomigration |   .9814353   .0925663    -0.20   0.843     .8157906    1.180714
  parents_evernottogether |   1.096427   .0897737     1.12   0.261     .9338665    1.287285
                    siblings |   1.324591   .1519698     2.45   0.014     1.057849    1.658593
                    demodiff |   1.211843   .1268961     1.83   0.067     .9869937    1.487917
                             |
                      cohort |
                1 1991-1993  |   1.080548   .1314741     0.64   0.524     .8512861    1.371554
                2 1981-1983  |          1   1.50e-16    -1.90   0.057            1           1
                3 1971-1973  |    .729798   .1230315    -1.87   0.062     .5244506    1.015549
                4 2001-2003  |   7.466375   7.888379     1.90   0.057      .941451    59.21365
                             |
                       house |   2.138142   .4690484     3.46   0.001     1.390935    3.286747
                       _rcs1 |   2.007073   .3332258     4.20   0.000     1.449579    2.778974
                       _rcs2 |   1.135341   .1260419     1.14   0.253     .9133324    1.411315
                       _rcs3 |   1.026009   .0534567     0.49   0.622     .9264076    1.136318
                       _rcs4 |   .9974849   .0400826    -0.06   0.950     .9219385    1.079222
                       _rcs5 |   1.012406   .0280819     0.44   0.657     .9588362    1.068969
                  _rcs_rela1 |   1.717607   .2993565     3.10   0.002     1.220592    2.417003
                  _rcs_rela2 |   1.133794   .1417435     1.00   0.315        .8874    1.448601
                  _rcs_rela3 |   1.111903   .0672636     1.75   0.080     .9875842    1.251871
                  _rcs_rela4 |   1.056274   .0446075     1.30   0.195     .9723651    1.147423
                  _rcs_rela5 |   .9866944   .0284839    -0.46   0.643      .932417    1.044131
                       _cons |   .0094029   .0037495   -11.70   0.000     .0043036    .0205442
------------------------------------------------------------------------------------------
Note: Estimates are transformed only in the first equation.
```

Linear combinations (presented in Table 3)

```
. lincom c.contactm_lag + c.contactm_lag#1.isced_rec, eform


 ( 1)  [xb]contactm_lag + [xb]1.isced_rec#c.contactm_lag = 0
```

```
------------------------------------------------------------------------------
             |     exp(b)   Std. err.      z    P>|z|     [95% conf. interval]
-------------+----------------------------------------------------------------
         (1) |   1.325839   .1791063     2.09   0.037     1.017426    1.727741
------------------------------------------------------------------------------

. lincom c.contactm_lag + c.contactm_lag#2.isced_rec, eform

 ( 1)  [xb]contactm_lag + [xb]2b.isced_rec#co.contactm_lag = 0

------------------------------------------------------------------------------
             |     exp(b)   Std. err.      z    P>|z|     [95% conf. interval]
-------------+----------------------------------------------------------------
         (1) |   1.132141   .0539378     2.61   0.009      1.03121     1.24295
------------------------------------------------------------------------------

. lincom c.contactm_lag + c.contactm_lag#3.isced_rec, eform

 ( 1)  [xb]contactm_lag + [xb]3.isced_rec#c.contactm_lag = 0

------------------------------------------------------------------------------
             |     exp(b)   Std. err.      z    P>|z|     [95% conf. interval]
-------------+----------------------------------------------------------------
         (1) |    .806095   .0495359    -3.51   0.000     .7146254    .9092725
------------------------------------------------------------------------------

. lincom c.contactm_lag + c.contactm_lag#4.isced_rec, eform

 ( 1)  [xb]contactm_lag + [xb]4.isced_rec#c.contactm_lag = 0

------------------------------------------------------------------------------
             |     exp(b)   Std. err.      z    P>|z|     [95% conf. interval]
-------------+----------------------------------------------------------------
         (1) |   1.353381   .2801094     1.46   0.144     .9020835    2.030455
------------------------------------------------------------------------------

. lincom c.contactf_lag + c.contactf_lag#1.isced_rec, eform

 ( 1)  [xb]contactf_lag + [xb]1.isced_rec#c.contactf_lag = 0
```

```
------------------------------------------------------------------------------
             |     exp(b)   Std. err.      z    P>|z|     [95% conf. interval]
-------------+----------------------------------------------------------------
         (1) |   .9366304   .0796853    -0.77   0.442     .7927768    1.106587
------------------------------------------------------------------------------

. lincom c.contactf_lag + c.contactf_lag#2.isced_rec, eform

 ( 1)  [xb]contactf_lag + [xb]2b.isced_rec#co.contactf_lag = 0

------------------------------------------------------------------------------
             |     exp(b)   Std. err.      z    P>|z|     [95% conf. interval]
-------------+----------------------------------------------------------------
         (1) |   1.079801   .0399228     2.08   0.038     1.004321    1.160953
------------------------------------------------------------------------------

. lincom c.contactf_lag + c.contactf_lag#3.isced_rec, eform

 ( 1)  [xb]contactf_lag + [xb]3.isced_rec#c.contactf_lag = 0

------------------------------------------------------------------------------
             |     exp(b)   Std. err.      z    P>|z|     [95% conf. interval]
-------------+----------------------------------------------------------------
         (1) |   1.205723   .0658349     3.43   0.001     1.083353    1.341915
------------------------------------------------------------------------------
. lincom c.contactf_lag + c.contactf_lag#4.isced_rec, eform

 ( 1)  [xb]contactf_lag + [xb]4.isced_rec#c.contactf_lag = 0

------------------------------------------------------------------------------
             |     exp(b)   Std. err.      z    P>|z|     [95% conf. interval]
-------------+----------------------------------------------------------------
         (1) |   .7659315   .0921056    -2.22   0.027     .6051045    .9695037
------------------------------------------------------------------------------
```

Model 4 (presented in Table 4)

```
. eststo isced4: stpm2 c.contactm_lag##b2.isced_rec##sex c.contactf_lag##b2.isced_rec##sex rela
german_native_nomigration parents_evernottogether siblings demodiff b2.cohort house, scale(ha

> zard) df(5) tvc(rela) dftvc(5) eform

note: delayed entry models are being fitted


Iteration 0:   log likelihood = -33.827316

Iteration 1:   log likelihood = -22.339113

Iteration 2:   log likelihood = -14.196698

Iteration 3:   log likelihood = -12.590625

Iteration 4:   log likelihood = -12.576508

Iteration 5:   log likelihood = -12.576503

Iteration 6:   log likelihood = -12.576503


Log likelihood = -12.576503                             Number of obs = 16,533


----------------------------------------------------------------------------------------------

                             |     exp(b)   Std. err.      z    P>|z|     [95% conf. interval]

-----------------------------+----------------------------------------------------------------

xb                           |

                contactm_lag |   1.022615   .0712794     0.32   0.748     .8920326    1.172313

                             |

                   isced_rec |

                          1  |   .6634377   .7092084    -0.38   0.701     .0816345    5.391707

                          3  |   2.113233    1.04319     1.52   0.130     .8030722    5.560836

                          4  |   .3847937   .6808636    -0.54   0.589     .0119975    12.34142

                             |

     isced_rec#c.contactm_lag |

                          1  |    1.14759   .2338053     0.68   0.499     .7697794    1.710831

                          2  |          1   1.20e-15     0.54   0.592            1           1

                          3  |   .7638275   .0854605    -2.41   0.016     .6134215    .9511119

                          4  |   1.148404   .4098259     0.39   0.698     .5705977    2.311316

                             |

                       1.sex |   .4058813    .209442    -1.75   0.081     .1476264    1.115923

                             |

           sex#c.contactm_lag |

                          0  |          1   1.27e-17    -2.42   0.016            1           1

                          1  |   1.196585   .1164994     1.84   0.065     .9887136    1.448159

                             |

               isced_rec#sex |
```

1 0	1	3.43e-16	-1.94	0.053	1	1
1 1	5.354844	9.157086	0.98	0.326	.1875617	152.8796
2 0	1	1.30e-16	1.62	0.105	1	1
3 1	2.604751	1.939769	1.29	0.199	.6051638	11.21139
4 0	1	6.15e-17	-1.07	0.285	1	1
4 1	6.235084	15.04948	0.76	0.448	.0549942	706.9155
isced_rec#sex#c.contactm_lag						
1 0	1	7.96e-17	-2.18	0.029	1	1
1 1	1.068905	.3194766	0.22	0.824	.5950183	1.920207
2 0	1	4.98e-16	-1.59	0.111	1	1
3 1	.9140598	.1429335	-0.57	0.566	.6727758	1.241878
4 0	1	6.86e-17	-1.96	0.050	1	1
4 1	.9763085	.4400793	-0.05	0.958	.4035532	2.361965
contactf_lag	1.07069	.0584051	1.25	0.211	.9621254	1.191505
isced_rec#c.contactf_lag						
1	1.04711	.1478593	0.33	0.744	.7939565	1.380982
2	1	7.34e-17	1.95	0.052	1	1
3	1.183449	.1129591	1.76	0.078	.9815289	1.426908
4	.8939576	.2227483	-0.45	0.653	.5485575	1.456839
sex#c.contactf_lag						
1	1.031517	.0735446	0.44	0.663	.8969905	1.186219
isced_rec#sex#c.contactf_lag						
1 0	1	8.33e-17	-1.88	0.060	1	1
1 1	.6481271	.1229554	-2.29	0.022	.4468685	.9400277
2 0	1	6.25e-17	1.92	0.054	1	1
3 1	.8701978	.1117012	-1.08	0.279	.6766362	1.11913
4 0	1	2.50e-16	1.92	0.054	1	1
4 1	.7075238	.2046983	-1.20	0.232	.4013049	1.247406
rela	9.613293	2.330051	9.34	0.000	5.978052	15.45912
german_native_nomigration	.9645466	.0913865	-0.38	0.703	.8010796	1.161371
parents_evernottogether	1.102734	.0906149	1.19	0.234	.9386961	1.295438
siblings	1.299074	.1492679	2.28	0.023	1.037118	1.627196
demodiff	1.219022	.1280125	1.89	0.059	.9922583	1.497609

```
                         |
                  cohort |
             1 1991-1993  |   1.058583   .1291591     0.47   0.641     .8334293    1.344563
             2 1981-1983  |          1   2.50e-16     1.92   0.055            1           1
             3 1971-1973  |    .727351   .1226717    -1.89   0.059     .5226179    1.012287
             4 2001-2003  |   7.642398     8.1054     1.92   0.055     .9560036    61.09418
                         |
                   house |   2.067857   .4569513     3.29   0.001     1.340983    3.188728
                   _rcs1 |   2.010809   .3306992     4.25   0.000     1.456738     2.77562
                   _rcs2 |   1.138665   .1264545     1.17   0.242     .9159372    1.415553
                   _rcs3 |   1.024168   .0540214     0.45   0.651     .9235776    1.135715
                   _rcs4 |   .9963197   .0401453    -0.09   0.927     .9206632    1.078193
                   _rcs5 |   1.011629   .0280893     0.42   0.677     .9580466    1.068209
              _rcs_rela1 |   1.729134   .2986599     3.17   0.002     1.232557    2.425772
              _rcs_rela2 |   1.137711    .142378     1.03   0.303     .8902443    1.453969
              _rcs_rela3 |   1.114893   .0682338     1.78   0.076     .9888669     1.25698
              _rcs_rela4 |    1.05652   .0447745     1.30   0.195     .9723093    1.148024
              _rcs_rela5 |    .986999   .0285258    -0.45   0.651     .9326434    1.044522
                   _cons |   .0165185   .0074681    -9.08   0.000     .0068098    .0400686
--------------------------------------------------------------------------------------------
Note: Estimates are transformed only in the first equation.
```

Linear combinations (presented in Table 5)

```
. lincom c.contactf_lag + c.contactf_lag#1.isced_rec + 0.sex, eform

 ( 1)  [xb]0b.sex + [xb]contactf_lag + [xb]1.isced_rec#c.contactf_lag = 0

------------------------------------------------------------------------------
             |     exp(b)   Std. err.      z    P>|z|     [95% conf. interval]
-------------+----------------------------------------------------------------
         (1) |   1.121131   .1473504     0.87   0.384      .866528     1.45054
------------------------------------------------------------------------------

. lincom c.contactf_lag + c.contactf_lag#2.isced_rec + 0.sex, eform

 ( 1)  [xb]0b.sex + [xb]contactf_lag + [xb]2b.isced_rec#co.contactf_lag = 0
```

```
------------------------------------------------------------------------------
             |     exp(b)   Std. err.      z    P>|z|     [95% conf. interval]
-------------+----------------------------------------------------------------
         (1) |    1.07069   .0584051     1.25   0.211     .9621254    1.191505
------------------------------------------------------------------------------

. lincom c.contactf_lag + c.contactf_lag#3.isced_rec + 0.sex, eform

 ( 1)  [xb]0b.sex + [xb]contactf_lag + [xb]3.isced_rec#c.contactf_lag = 0

------------------------------------------------------------------------------
             |     exp(b)   Std. err.      z    P>|z|     [95% conf. interval]
-------------+----------------------------------------------------------------
         (1) |   1.267107   .1007463     2.98   0.003     1.084264    1.480783
------------------------------------------------------------------------------

. lincom c.contactf_lag + c.contactf_lag#4.isced_rec + 0.sex, eform

 ( 1)  [xb]0b.sex + [xb]contactf_lag + [xb]4.isced_rec#c.contactf_lag = 0

------------------------------------------------------------------------------
             |     exp(b)   Std. err.      z    P>|z|     [95% conf. interval]
-------------+----------------------------------------------------------------
         (1) |   .9571517   .2333221    -0.18   0.857     .5935889     1.54339
------------------------------------------------------------------------------

.
. lincom c.contactf_lag + c.contactf_lag#1.isced_rec + 1.sex, eform

 ( 1)  [xb]1.sex + [xb]contactf_lag + [xb]1.isced_rec#c.contactf_lag = 0

------------------------------------------------------------------------------
             |     exp(b)   Std. err.      z    P>|z|     [95% conf. interval]
-------------+----------------------------------------------------------------
         (1) |   .4550459   .2423711    -1.48   0.139     .1602064      1.2925
------------------------------------------------------------------------------

. lincom c.contactf_lag + c.contactf_lag#2.isced_rec + 1.sex, eform
```

```
 ( 1)  [xb]1.sex + [xb]contactf_lag + [xb]2b.isced_rec#co.contactf_lag = 0

------------------------------------------------------------------------------
             |     exp(b)   Std. err.      z    P>|z|     [95% conf. interval]
-------------+----------------------------------------------------------------
         (1) |   .4345732    .228129    -1.59   0.112     .1553191    1.215908
------------------------------------------------------------------------------

. lincom c.contactf_lag + c.contactf_lag#3.isced_rec + 1.sex, eform

 ( 1)  [xb]1.sex + [xb]contactf_lag + [xb]3.isced_rec#c.contactf_lag = 0

------------------------------------------------------------------------------
             |     exp(b)   Std. err.      z    P>|z|     [95% conf. interval]
-------------+----------------------------------------------------------------
         (1) |   .5142951   .2683189    -1.27   0.202     .1849788     1.42989
------------------------------------------------------------------------------

. lincom c.contactf_lag + c.contactf_lag#4.isced_rec + 1.sex, eform

 ( 1)  [xb]1.sex + [xb]contactf_lag + [xb]4.isced_rec#c.contactf_lag = 0

------------------------------------------------------------------------------
             |     exp(b)   Std. err.      z    P>|z|     [95% conf. interval]
-------------+----------------------------------------------------------------
         (1) |     .38849   .2215266    -1.66   0.097     .1270585    1.187835
------------------------------------------------------------------------------
```

Online Supplementary Material C

Parents aged 66+

Model 3 (presented in Table 2)

```
. eststo isced3: stpm2 c.contactm_lag##b3.isced_rec c.contactf_lag##b3.isced_rec sex rela
german_native_nomigration parents_evernottogether siblings demodiff b2.cohort father_66 mother_66,

> scale(hazard) df(5) tvc(rela) dftvc(5) eform

note: delayed entry models are being fitted


Iteration 0:   log likelihood = -20.282306

Iteration 1:   log likelihood = -11.850563

Iteration 2:   log likelihood = -4.5280273

Iteration 3:   log likelihood = -2.8646162

Iteration 4:   log likelihood = -2.8465065

Iteration 5:   log likelihood = -2.8433792

Iteration 6:   log likelihood = -2.8428666

Iteration 7:   log likelihood = -2.8427431

Iteration 8:   log likelihood = -2.8427168

Iteration 9:   log likelihood = -2.8427113

Iteration 10:  log likelihood =   -2.84271

Iteration 11:  log likelihood = -2.8427097


Log likelihood = -2.8427097                           Number of obs = 14,990


------------------------------------------------------------------------------------------

                          |     exp(b)   Std. err.      z    P>|z|     [95% conf. interval]

--------------------------+---------------------------------------------------------------

xb                        |

             contactm_lag |   .8195742    .0536717    -3.04   0.002     .7208509    .9318181

                          |

                isced_rec |

                        1 |   .7508581    .6725793    -0.32   0.749     .1297467    4.345298

                        2 |   .2393367    .0937539    -3.65   0.000      .111064     .515757

                        3 |          1    1.92e-13   -0.00   0.997            1           1

                        4 |   .1841415    .2464021    -1.26   0.206     .0133705    2.536037

                          |

  isced_rec#c.contactm_lag |

                        1 |   1.428133    .2256675     2.26   0.024     1.047769    1.946578
```

2	1.401074	.1167082	4.05	0.000	1.190027	1.649549
3	1	4.51e-13	-0.00	0.997	1	1
4	1.904521	.4512472	2.72	0.007	1.197034	3.030156
contactf_lag	1.185165	.071049	2.83	0.005	1.053782	1.33293
isced_rec#c.contactf_lag						
1	.8332332	.0997271	-1.52	0.127	.6590053	1.053524
2	.9207086	.0664087	-1.15	0.252	.7993314	1.060517
4	.5970839	.0850836	-3.62	0.000	.4515858	.7894604
sex	1.18639	.0851901	2.38	0.017	1.030637	1.36568
rela	9.752895	2.557653	8.68	0.000	5.833238	16.30637
german_native_nomigration	.993892	.0989283	-0.06	0.951	.8177373	1.207993
parents_evernottogether	1.104992	.0940504	1.17	0.241	.9352115	1.305595
siblings	1.345114	.1605951	2.48	0.013	1.064469	1.699751
demodiff	1.124927	.1247682	1.06	0.289	.9051402	1.398084
cohort						
1 1991-1993	1.061464	.1347011	0.47	0.638	.827726	1.361206
3 1971-1973	.6579073	.120999	-2.28	0.023	.4587921	.9434381
4 2001-2003	5.02e-07	.0021631	-0.00	0.997	0	.
father_66	.8966366	.0929282	-1.05	0.292	.7318081	1.09859
mother_66	.9095689	.1315845	-0.66	0.512	.6850068	1.207748
_rcs1	2.076122	.3761555	4.03	0.000	1.455562	2.96125
_rcs2	1.13072	.1355438	1.02	0.305	.8939598	1.430184
_rcs3	1.024799	.059046	0.43	0.671	.9153659	1.147314
_rcs4	.9862729	.0429783	-0.32	0.751	.9055341	1.074211
_rcs5	1.010339	.0313734	0.33	0.740	.9506824	1.07374
_rcs_rela1	1.719357	.3274819	2.85	0.004	1.183695	2.497425
_rcs_rela2	1.13174	.1520377	0.92	0.357	.869754	1.472641
_rcs_rela3	1.109387	.072702	1.58	0.113	.9756658	1.261437
_rcs_rela4	1.064078	.0485988	1.36	0.174	.9729645	1.163723
_rcs_rela5	.989876	.0318857	-0.32	0.752	.929313	1.054386
_cons	.033235	.0140262	-8.07	0.000	.0145332	.0760028

Note: Estimates are transformed only in the first equation.

Linear combinations (presented in Table 3)

```
. lincom c.contactm_lag + c.contactm_lag#1.isced_rec, eform

 ( 1)  [xb]contactm_lag + [xb]1.isced_rec#c.contactm_lag = 0

------------------------------------------------------------------------------
             |     exp(b)   Std. err.      z    P>|z|     [95% conf. interval]
-------------+----------------------------------------------------------------
         (1) |   1.170461   .1684572     1.09   0.274     .8827723    1.551905
------------------------------------------------------------------------------

. lincom c.contactm_lag + c.contactm_lag#2.isced_rec, eform

 ( 1)  [xb]contactm_lag + [xb]2.isced_rec#c.contactm_lag = 0

------------------------------------------------------------------------------
             |     exp(b)   Std. err.      z    P>|z|     [95% conf. interval]
-------------+----------------------------------------------------------------
         (1) |   1.148284    .060626     2.62   0.009       1.0354    1.273474
------------------------------------------------------------------------------

. lincom c.contactm_lag + c.contactm_lag#3.isced_rec, eform

 ( 1)  [xb]contactm_lag + [xb]3b.isced_rec#co.contactm_lag = 0

------------------------------------------------------------------------------
             |     exp(b)   Std. err.      z    P>|z|     [95% conf. interval]
-------------+----------------------------------------------------------------
         (1) |   .8195742   .0536717    -3.04   0.002     .7208509    .9318181
------------------------------------------------------------------------------

. lincom c.contactm_lag + c.contactm_lag#4.isced_rec, eform

 ( 1)  [xb]contactm_lag + [xb]4.isced_rec#c.contactm_lag = 0

------------------------------------------------------------------------------
             |     exp(b)   Std. err.      z    P>|z|     [95% conf. interval]
-------------+----------------------------------------------------------------
```

```
        (1) |   1.560896   .3561162     1.95   0.051     .9980989    2.441037
------------------------------------------------------------------------------

.
. lincom c.contactf_lag + c.contactf_lag#1.isced_rec, eform

 ( 1)  [xb]contactf_lag + [xb]1.isced_rec#c.contactf_lag = 0

------------------------------------------------------------------------------
             |     exp(b)   Std. err.      z    P>|z|     [95% conf. interval]
-------------+----------------------------------------------------------------
        (1) |   .9875191   .1032759    -0.12   0.904     .8044997    1.212174
------------------------------------------------------------------------------

. lincom c.contactf_lag + c.contactf_lag#2.isced_rec, eform

 ( 1)  [xb]contactf_lag + [xb]2.isced_rec#c.contactf_lag = 0

------------------------------------------------------------------------------
             |     exp(b)   Std. err.      z    P>|z|     [95% conf. interval]
-------------+----------------------------------------------------------------
        (1) |   1.091192   .0475727     2.00   0.045     1.001824    1.188532
------------------------------------------------------------------------------

. lincom c.contactf_lag + c.contactf_lag#3.isced_rec, eform

 ( 1)  [xb]contactf_lag + [xb]3b.isced_rec#co.contactf_lag = 0

------------------------------------------------------------------------------
             |     exp(b)   Std. err.      z    P>|z|     [95% conf. interval]
-------------+----------------------------------------------------------------
        (1) |   1.185165    .071049     2.83   0.005     1.053782     1.33293
------------------------------------------------------------------------------

. lincom c.contactf_lag + c.contactf_lag#4.isced_rec, eform

 ( 1)  [xb]contactf_lag + [xb]4.isced_rec#c.contactf_lag = 0

------------------------------------------------------------------------------
```

```
             |     exp(b)   Std. err.      z    P>|z|     [95% conf. interval]
-------------+----------------------------------------------------------------
         (1) |   .7076431   .0923819    -2.65   0.008      .547887    .9139819
------------------------------------------------------------------------------
```

Model 4 (presented in Table 4)

```
. eststo isced4: stpm2 c.contactm_lag##b2.isced_rec##sex c.contactf_lag##b2.isced_rec##sex rela
german_native_nomigration parents_evernottogether siblings demodiff b2.cohort father_66 mothe
> r_66, scale(hazard) df(5) tvc(rela) dftvc(5) eform
note: delayed entry models are being fitted

Iteration 0:   log likelihood = -11.790659
Iteration 1:   log likelihood = -4.1061863
Iteration 2:   log likelihood =   2.398014
Iteration 3:   log likelihood =  5.3558843
Iteration 4:   log likelihood =  5.5073864
Iteration 5:   log likelihood =  5.5114619
Iteration 6:   log likelihood =  5.5117537
Iteration 7:   log likelihood =  5.5118165
Iteration 8:   log likelihood =  5.5118296
Iteration 9:   log likelihood =  5.5118325
Iteration 10:  log likelihood =  5.5118332
Iteration 11:  log likelihood =  5.5118334

Log likelihood = 5.5118334                             Number of obs = 14,990

---------------------------------------------------------------------------------------------
                             |     exp(b)   Std. err.      z    P>|z|     [95% conf. interval]
-----------------------------+---------------------------------------------------------------
xb                           |
                contactm_lag |   1.050575   .0871591     0.59   0.552     .8929116    1.236077
                             |
                   isced_rec |
                          1  |   2.306673   2.616009     0.74   0.461     .2498239    21.29796
                          3  |   2.081202   1.119563     1.36   0.173     .7251346     5.97324
                          4  |   .1833694   .3835534    -0.81   0.417       .00304    11.06071
                             |
    isced_rec#c.contactm_lag |
                          1  |   .9317332    .202725    -0.32   0.745     .6082589    1.427232
                          2  |          1   2.94e-16    -0.79   0.431            1           1
```

3	.7472837	.0946886	-2.30	0.022	.5829474	.9579472
4	1.362034	.5868195	0.72	0.473	.5854045	3.168985
1.sex	.3696421	.2077778	-1.77	0.077	.1228334	1.112363
sex#c.contactm_lag						
0	1	3.02e-13	0.00	0.998	1	1
1	1.16137	.1272393	1.37	0.172	.9369435	1.439554
isced_rec#sex						
1 0	1	3.36e-13	-0.00	0.998	1	1
1 1	2.395733	4.500218	0.47	0.642	.0603276	95.13959
2 0	1	1.79e-13	0.00	0.998	1	1
3 1	3.328703	2.64059	1.52	0.130	.7031288	15.75851
4 0	1	6.55e-13	-0.00	0.998	1	1
4 1	13.9089	37.8019	0.97	0.333	.0675897	2862.236
isced_rec#sex#c.contactm_lag						
1 0	1	4.44e-14	-0.01	0.994	1	1
1 1	1.243551	.4100382	0.66	0.509	.6516212	2.373186
2 0	1	5.47e-13	0.00	0.998	1	1
3 1	.9589074	.1638684	-0.25	0.806	.6859843	1.340415
4 0	1	8.75e-13	0.00	0.998	1	1
4 1	.903139	.4707929	-0.20	0.845	.3251142	2.508842
contactf_lag	1.054442	.071466	0.78	0.434	.9232756	1.204242
isced_rec#c.contactf_lag						
1	1.033297	.1784991	0.19	0.850	.7365166	1.449666
2	1	3.56e-13	0.00	0.998	1	1
3	1.213997	.1372924	1.71	0.086	.9726443	1.515238
4	.8612632	.2673292	-0.48	0.630	.4687375	1.582494
sex#c.contactf_lag						
1	1.082386	.0938252	0.91	0.361	.9132652	1.282825
isced_rec#sex#c.contactf_lag						
1 0	1	2.19e-13	-0.00	0.998	1	1
1 1	.7136617	.1691762	-1.42	0.155	.4484477	1.135724

```
                    2 0  |          1   2.19e-13    -0.00   0.998            1           1
                    3 1  |   .7927662   .1165804    -1.58   0.114     .5942531    1.057593
                    4 0  |          1   1.20e-12    -0.00   0.998            1           1
                    4 1  |   .6669856   .2319896    -1.16   0.244     .3373287    1.318802
                         |
                    rela |   9.546069   2.495591     8.63   0.000     5.718714    15.93495
german_native_nomigration |  .9620856   .0960454    -0.39   0.699     .7911119     1.17001
 parents_evernottogether |   1.110162   .0950847     1.22   0.222     .9386017     1.31308
                siblings |   1.341001   .1606367     2.45   0.014     1.060388    1.695873
                demodiff |   1.137253    .126427     1.16   0.247     .9145979    1.414113
                         |
                  cohort |
             1 1991-1993 |   1.045053   .1331347     0.35   0.729     .8141405    1.341458
             2 1981-1983 |          1   4.38e-13     0.00   0.998            1           1
             3 1971-1973 |   .6499814   .1198516    -2.34   0.019     .4528412    .9329448
             4 2001-2003 |   2.79e-07    .001557    -0.00   0.998            0           .
                         |
               father_66 |   .8986426   .0931489    -1.03   0.303     .7334247    1.101079
               mother_66 |   .9017789   .1304182    -0.71   0.475     .6791983    1.197302
                   _rcs1 |   2.073568    .374275     4.04   0.000      1.45572    2.953646
                   _rcs2 |   1.130605    .135512     1.02   0.306     .8938968    1.429993
                   _rcs3 |   1.022639   .0592269     0.39   0.699     .9129025    1.145567
                   _rcs4 |   .9850006   .0429002    -0.35   0.729     .9044066    1.072777
                   _rcs5 |   1.009549   .0313301     0.31   0.759     .9499733    1.072861
              _rcs_rela1 |   1.742977   .3304988     2.93   0.003     1.201957    2.527518
              _rcs_rela2 |   1.140023   .1531553     0.98   0.329     .8761119    1.483431
              _rcs_rela3 |   1.110953    .073287     1.60   0.111     .9762118    1.264293
              _rcs_rela4 |   1.064906    .048658     1.38   0.169     .9736836    1.164675
              _rcs_rela5 |     .99049   .0319062    -0.30   0.767     .9298882    1.055041
                   _cons |   .0152892   .0076365    -8.37   0.000     .0057443    .0406944
----------------------------------------------------------------------------------------------
Note: Estimates are transformed only in the first equation.
```

Linear combinations (presented in Table 5)

```
. lincom c.contactf_lag + c.contactf_lag#1.isced_rec + 0.sex, eform


 ( 1)  [xb]0b.sex + [xb]contactf_lag + [xb]1.isced_rec#c.contactf_lag = 0
```

```
------------------------------------------------------------------------------
             |     exp(b)   Std. err.      z    P>|z|     [95% conf. interval]
-------------+----------------------------------------------------------------
         (1) |   1.089551   .1738137     0.54   0.591     .7969983    1.489491
------------------------------------------------------------------------------

. lincom c.contactf_lag + c.contactf_lag#2.isced_rec + 0.sex, eform

 ( 1)  [xb]0b.sex + [xb]contactf_lag + [xb]2b.isced_rec#co.contactf_lag = 0

------------------------------------------------------------------------------
             |     exp(b)   Std. err.      z    P>|z|     [95% conf. interval]
-------------+----------------------------------------------------------------
         (1) |   1.054442    .071466     0.78   0.434     .9232756    1.204242
------------------------------------------------------------------------------

. lincom c.contactf_lag + c.contactf_lag#3.isced_rec + 0.sex, eform

 ( 1)  [xb]0b.sex + [xb]contactf_lag + [xb]3.isced_rec#c.contactf_lag = 0

------------------------------------------------------------------------------
             |     exp(b)   Std. err.      z    P>|z|     [95% conf. interval]
-------------+----------------------------------------------------------------
         (1) |   1.280089   .1171126     2.70   0.007     1.069955    1.531492
------------------------------------------------------------------------------

. lincom c.contactf_lag + c.contactf_lag#4.isced_rec + 0.sex, eform

 ( 1)  [xb]0b.sex + [xb]contactf_lag + [xb]4.isced_rec#c.contactf_lag = 0

------------------------------------------------------------------------------
             |     exp(b)   Std. err.      z    P>|z|     [95% conf. interval]
-------------+----------------------------------------------------------------
         (1) |   .9081517   .2755064    -0.32   0.751     .5011052    1.645841
------------------------------------------------------------------------------

.
. lincom c.contactf_lag + c.contactf_lag#1.isced_rec + 1.sex, eform
```

```
 ( 1)  [xb]1.sex + [xb]contactf_lag + [xb]1.isced_rec#c.contactf_lag = 0

------------------------------------------------------------------------------
             |     exp(b)   Std. err.      z    P>|z|     [95% conf. interval]
-------------+----------------------------------------------------------------
         (1) |   .4027441    .235014    -1.56   0.119     .1283293    1.263957
------------------------------------------------------------------------------

. lincom c.contactf_lag + c.contactf_lag#2.isced_rec + 1.sex, eform

 ( 1)  [xb]1.sex + [xb]contactf_lag + [xb]2b.isced_rec#co.contactf_lag = 0

------------------------------------------------------------------------------
             |     exp(b)   Std. err.      z    P>|z|     [95% conf. interval]
-------------+----------------------------------------------------------------
         (1) |    .389766   .2237886    -1.64   0.101     .1264961    1.200967
------------------------------------------------------------------------------

. lincom c.contactf_lag + c.contactf_lag#3.isced_rec + 1.sex, eform

 ( 1)  [xb]1.sex + [xb]contactf_lag + [xb]3.isced_rec#c.contactf_lag = 0

------------------------------------------------------------------------------
             |     exp(b)   Std. err.      z    P>|z|     [95% conf. interval]
-------------+----------------------------------------------------------------
         (1) |   .4731747   .2692643    -1.31   0.189     .1551092    1.443462
------------------------------------------------------------------------------

. lincom c.contactf_lag + c.contactf_lag#4.isced_rec + 1.sex, eform

 ( 1)  [xb]1.sex + [xb]contactf_lag + [xb]4.isced_rec#c.contactf_lag = 0

------------------------------------------------------------------------------
             |     exp(b)   Std. err.      z    P>|z|     [95% conf. interval]
-------------+----------------------------------------------------------------
         (1) |   .3356911   .2142551    -1.71   0.087      .096086    1.172789
------------------------------------------------------------------------------
```

Online Supplementary Material D

Including indicators for vulnerable parents

Model 3 (presented in Table 2)

```
. eststo isced3: stpm2 c.contactm_lag##b2.isced_rec c.contactf_lag##b2.isced_rec sex rela
german_native_nomigration parents_evernottogether siblings demodiff b2.cohort vulnerable_f vulnerab

> le_m, scale(hazard) df(5) tvc(rela) dftvc(5) eform

note: delayed entry models are being fitted


Iteration 0:   log likelihood = -23.091823

Iteration 1:   log likelihood = -14.531005

Iteration 2:   log likelihood = -3.2559492

Iteration 3:   log likelihood = -1.1529918

Iteration 4:   log likelihood = -1.1339652

Iteration 5:   log likelihood = -1.1339582

Iteration 6:   log likelihood = -1.1339582


Log likelihood = -1.1339582                             Number of obs = 15,292


------------------------------------------------------------------------------------------
                          |     exp(b)   Std. err.      z    P>|z|     [95% conf. interval]
--------------------------+---------------------------------------------------------------
xb                        |
             contactm_lag |   1.139346   .0567859     2.62   0.009     1.033311    1.256262
                          |
                isced_rec |
                        1 |   1.624828   1.409063     0.56   0.576     .2969195     8.89152
                        3 |   4.066612   1.531906     3.72   0.000      1.94349    8.509091
                        4 |   .9612772    1.18351    -0.03   0.974      .086072    10.73582
                          |
 isced_rec#c.contactm_lag |
                        1 |   1.224056   .1947883     1.27   0.204     .8960801    1.672076
                        2 |          1   4.05e-16    -0.10   0.924            1           1
                        3 |   .7126941   .0567093    -4.26   0.000     .6097795    .8329779
                        4 |    1.22544   .2662207     0.94   0.349     .8005215    1.875907
                          |
             contactf_lag |   1.097095   .0423443     2.40   0.016     1.017163    1.183308
                          |
```

```
 isced_rec#c.contactf_lag |
                      1  |   .8174692   .0794394    -2.07   0.038     .6757001     .988983
                      3  |   1.099161   .0727047     1.43   0.153     .9655127    1.251309
                      4  |   .6876311   .0874784    -2.94   0.003     .5358807     .882354
                         |
                      sex |    1.11394   .0782235     1.54   0.124     .9707072    1.278307
                     rela |   9.394179   2.419575     8.70   0.000     5.670514    15.56307
german_native_nomigration |   .9385109   .0901829    -0.66   0.509     .7774029    1.133007
  parents_evernottogether |   1.136149   .0965246     1.50   0.133     .9618759    1.341996
                 siblings |    1.26517   .1463781     2.03   0.042     1.008477    1.587199
                 demodiff |     1.1872   .1256887     1.62   0.105     .9647337    1.460968
                          |
                   cohort |
             1 1991-1993  |   1.209317   .1594374     1.44   0.149     .9339354    1.565897
             2 1981-1983  |          1   2.44e-17     0.12   0.905            1           1
             3 1971-1973  |   .6962786     .12249    -2.06   0.040      .493218    .9829404
                          |
             vulnerable_f |   .9795626    .168862    -0.12   0.905     .6987111    1.373304
             vulnerable_m |   .9869839   .1667334    -0.08   0.938     .7087854    1.374376
                    _rcs1 |   1.996922   .3472519     3.98   0.000     1.420178    2.807887
                    _rcs2 |   1.129928   .1219634     1.13   0.258     .9144771    1.396139
                    _rcs3 |   1.031197   .0505196     0.63   0.531     .9367858    1.135123
                    _rcs4 |   .9789085   .0399987    -0.52   0.602     .9035695    1.060529
                    _rcs5 |   1.007218   .0300523     0.24   0.810      .950006    1.067876
               _rcs_rela1 |   1.819033   .3315117     3.28   0.001      1.27266    2.599972
               _rcs_rela2 |   1.170071   .1430141     1.29   0.199     .9208157    1.486798
               _rcs_rela3 |   1.111302   .0642894     1.82   0.068     .9921783    1.244729
               _rcs_rela4 |    1.06688   .0459965     1.50   0.133     .9804322     1.16095
               _rcs_rela5 |    .993525   .0308163    -0.21   0.834     .9349254    1.055797
                    _cons |    .009004   .0037428   -11.33   0.000     .0039866    .0203358
------------------------------------------------------------------------------------------
Note: Estimates are transformed only in the first equation.
```

Linear combinations (presented in Table 3)

```
. lincom c.contactm_lag + c.contactm_lag#1.isced_rec, eform


 ( 1)  [xb]contactm_lag + [xb]1.isced_rec#c.contactm_lag = 0
```

```
------------------------------------------------------------------------------
             |     exp(b)   Std. err.      z    P>|z|     [95% conf. interval]
-------------+----------------------------------------------------------------
         (1) |   1.394624   .2112276     2.20   0.028     1.036419     1.87663
------------------------------------------------------------------------------

. lincom c.contactm_lag + c.contactm_lag#2.isced_rec, eform

 ( 1)  [xb]contactm_lag + [xb]2b.isced_rec#co.contactm_lag = 0

------------------------------------------------------------------------------
             |     exp(b)   Std. err.      z    P>|z|     [95% conf. interval]
-------------+----------------------------------------------------------------
         (1) |   1.139346   .0567859     2.62   0.009     1.033311    1.256262
------------------------------------------------------------------------------

. lincom c.contactm_lag + c.contactm_lag#3.isced_rec, eform

 ( 1)  [xb]contactm_lag + [xb]3.isced_rec#c.contactm_lag = 0

------------------------------------------------------------------------------
             |     exp(b)   Std. err.      z    P>|z|     [95% conf. interval]
-------------+----------------------------------------------------------------
         (1) |   .8120051   .0512399    -3.30   0.001      .717539     .918908
------------------------------------------------------------------------------

. lincom c.contactm_lag + c.contactm_lag#4.isced_rec, eform

 ( 1)  [xb]contactm_lag + [xb]4.isced_rec#c.contactm_lag = 0

------------------------------------------------------------------------------
             |     exp(b)   Std. err.      z    P>|z|     [95% conf. interval]
-------------+----------------------------------------------------------------
         (1) |     1.3962   .2958418     1.58   0.115     .9216927    2.114995
------------------------------------------------------------------------------

. lincom c.contactf_lag + c.contactf_lag#1.isced_rec, eform

 ( 1)  [xb]contactf_lag + [xb]1.isced_rec#c.contactf_lag = 0
```

```
-----------------------------------------------------------------------------
             |     exp(b)   Std. err.      z    P>|z|     [95% conf. interval]
-------------+---------------------------------------------------------------
         (1) |   .8968416   .0817582    -1.19   0.232     .7500982    1.072293
-----------------------------------------------------------------------------

. lincom c.contactf_lag + c.contactf_lag#2.isced_rec, eform

 ( 1)  [xb]contactf_lag + [xb]2b.isced_rec#co.contactf_lag = 0

-----------------------------------------------------------------------------
             |     exp(b)   Std. err.      z    P>|z|     [95% conf. interval]
-------------+---------------------------------------------------------------
         (1) |   1.097095   .0423443     2.40   0.016     1.017163    1.183308
-----------------------------------------------------------------------------

. lincom c.contactf_lag + c.contactf_lag#3.isced_rec, eform

 ( 1)  [xb]contactf_lag + [xb]3.isced_rec#c.contactf_lag = 0

-----------------------------------------------------------------------------
             |     exp(b)   Std. err.      z    P>|z|     [95% conf. interval]
-------------+---------------------------------------------------------------
         (1) |   1.205884    .067903     3.32   0.001     1.079878    1.346593
-----------------------------------------------------------------------------

. lincom c.contactf_lag + c.contactf_lag#4.isced_rec, eform

 ( 1)  [xb]contactf_lag + [xb]4.isced_rec#c.contactf_lag = 0

-----------------------------------------------------------------------------
             |     exp(b)   Std. err.      z    P>|z|     [95% conf. interval]
-------------+---------------------------------------------------------------
         (1) |   .7543968    .092734    -2.29   0.022      .592879    .9599167
-----------------------------------------------------------------------------
```

Model 4 (presented in Table 4)

```
. eststo isced4: stpm2 c.contactm_lag##b2.isced_rec##sex c.contactf_lag##b2.isced_rec##sex rela
german_native_nomigration parents_evernottogether siblings demodiff b2.cohort vulnerable_f vu

> lnerable_m, scale(hazard) df(5) tvc(rela) dftvc(5) eform

note: delayed entry models are being fitted


Iteration 0:   log likelihood = -15.223595

Iteration 1:   log likelihood = -6.1451031

Iteration 2:   log likelihood =   4.828425

Iteration 3:   log likelihood =  6.8901292

Iteration 4:   log likelihood =  6.9075179

Iteration 5:   log likelihood =  6.9075231

Iteration 6:   log likelihood =  6.9075231


Log likelihood = 6.9075231                              Number of obs = 15,292


----------------------------------------------------------------------------------------------

                             |     exp(b)   Std. err.      z    P>|z|     [95% conf. interval]

-----------------------------+----------------------------------------------------------------

xb                           |

                contactm_lag |   1.038794   .0748518     0.53   0.597     .9019758    1.196366

                             |

                   isced_rec |

                          1  |    .669697   .7684792    -0.35   0.727     .0706516    6.347966

                          3  |   2.211435   1.122604     1.56   0.118     .8176616    5.981012

                          4  |   .3508921    .634422    -0.58   0.562     .0101433    12.13858

                             |

      isced_rec#c.contactm_lag |

                          1  |   1.225692   .2910315     0.86   0.391     .7696088    1.952059

                          2  |          1   1.23e-15     0.58   0.563            1           1

                          3  |   .7778899   .0902428    -2.17   0.030     .6196843    .9764855

                          4  |   1.180939      .4264     0.46   0.645     .5819497    2.396457

                             |

                       1.sex |   .4057158   .2181686    -1.68   0.093     .1414161    1.163978

                             |

            sex#c.contactm_lag |

                          0  |          1   1.11e-17    -1.27   0.205            1           1

                          1  |   1.178188   .1190297     1.62   0.105      .966539    1.436182

                             |

               isced_rec#sex |
```

1 0	1	5.76e-17	-0.23	0.818	1	1
1 1	10.25435	18.24609	1.31	0.191	.3135607	335.3471
2 0	1	3.44e-17	-0.74	0.457	1	1
3 1	3.067712	2.349327	1.46	0.143	.6838237	13.76211
4 0	1	1.35e-17	4.00	0.000	1	1
4 1	7.334766	18.07773	0.81	0.419	.0585411	918.9926
isced_rec#sex#c.contactm_lag						
1 0	1	1.50e-17	-1.47	0.141	1	1
1 1	.9182977	.2997304	-0.26	0.794	.4843431	1.74106
2 0	1	8.82e-17	1.69	0.092	1	1
3 1	.882924	.142924	-0.77	0.442	.6428867	1.212585
4 0	1	2.84e-17	-0.52	0.601	1	1
4 1	.9895978	.453292	-0.02	0.982	.4032367	2.428608
contactf_lag	1.082609	.0606923	1.42	0.157	.9699564	1.208345
isced_rec#c.contactf_lag						
1	.9945412	.1585722	-0.03	0.973	.7276207	1.359379
2	1	1.54e-17	0.23	0.819	1	1
3	1.159497	.1135424	1.51	0.131	.9570103	1.404826
4	.8887674	.2167865	-0.48	0.629	.5510165	1.433546
sex#c.contactf_lag						
1	1.040584	.0768017	0.54	0.590	.9004366	1.202545
isced_rec#sex#c.contactf_lag						
1 0	1	1.41e-17	0.26	0.795	1	1
1 1	.6821548	.1384554	-1.88	0.059	.4582648	1.015428
2 0	1	1.03e-17	0.04	0.967	1	1
3 1	.8738734	.1163372	-1.01	0.311	.6731774	1.134403
4 0	1	4.10e-17	0.04	0.967	1	1
4 1	.6649279	.1918128	-1.41	0.157	.3777697	1.170367
rela	9.341107	2.380813	8.77	0.000	5.668244	15.39388
german_native_nomigration	.9253442	.0895301	-0.80	0.423	.7655027	1.118562
parents_evernottogether	1.14285	.097504	1.57	0.118	.9668691	1.350861
siblings	1.243535	.1439962	1.88	0.060	.9910425	1.560356
demodiff	1.19625	.1270562	1.69	0.092	.9714356	1.473092

```
                  |
           cohort |
      1 1991-1993  |   1.199953   .1586023     1.38   0.168     .9260999    1.554787
      2 1981-1983  |          1   4.07e-17     0.02   0.984            1           1
      3 1971-1973  |   .6910479   .1214919    -2.10   0.036     .4896209    .9753407
                  |
     vulnerable_f |   1.003473   .1733956     0.02   0.984     .7151909    1.407957
     vulnerable_m |   .9588287   .1622616    -0.25   0.804     .6881658    1.335946
            _rcs1 |   2.003549   .3457566     4.03   0.000     1.428586    2.809917
            _rcs2 |   1.131611   .1221308     1.15   0.252     .9158622    1.398184
            _rcs3 |   1.030254   .0510838     0.60   0.548     .9348431    1.135403
            _rcs4 |   .9781849   .0401238    -0.54   0.591     .9026219    1.060074
            _rcs5 |   1.006482   .0301633     0.22   0.829     .9490657    1.067372
       _rcs_rela1 |   1.831586   .3313205     3.35   0.001     1.284846    2.610979
       _rcs_rela2 |    1.17474   .1437028     1.32   0.188      .924308    1.493025
       _rcs_rela3 |   1.114386    .065196     1.85   0.064     .9936578    1.249782
       _rcs_rela4 |   1.066802   .0462023     1.49   0.135     .9799838    1.161311
       _rcs_rela5 |    .993885   .0309672    -0.20   0.844     .9350065    1.056471
            _cons |   .0152974   .0072029    -8.88   0.000     .0060789    .0384959
-----------------------------------------------------------------------------------------------
Note: Estimates are transformed only in the first equation.
```

Linear combinations (presented in Table 5)

```
. lincom c.contactf_lag + c.contactf_lag#1.isced_rec + 0.sex, eform

 ( 1)  [xb]0b.sex + [xb]contactf_lag + [xb]1.isced_rec#c.contactf_lag = 0

------------------------------------------------------------------------------
             |     exp(b)   Std. err.      z    P>|z|     [95% conf. interval]
-------------+----------------------------------------------------------------
         (1) |   1.076699    .162095     0.49   0.624     .8015813    1.446242
------------------------------------------------------------------------------

. lincom c.contactf_lag + c.contactf_lag#2.isced_rec + 0.sex, eform

 ( 1)  [xb]0b.sex + [xb]contactf_lag + [xb]2b.isced_rec#co.contactf_lag = 0

------------------------------------------------------------------------------
```

```
             |     exp(b)   Std. err.      z    P>|z|     [95% conf. interval]
-------------+----------------------------------------------------------------
         (1) |   1.082609   .0606923     1.42   0.157     .9699564    1.208345
------------------------------------------------------------------------------

. lincom c.contactf_lag + c.contactf_lag#3.isced_rec + 0.sex, eform

 ( 1)  [xb]0b.sex + [xb]contactf_lag + [xb]3.isced_rec#c.contactf_lag = 0

------------------------------------------------------------------------------
             |     exp(b)   Std. err.      z    P>|z|     [95% conf. interval]
-------------+----------------------------------------------------------------
         (1) |   1.255281    .102263     2.79   0.005     1.070032    1.472602
------------------------------------------------------------------------------

. lincom c.contactf_lag + c.contactf_lag#4.isced_rec + 0.sex, eform

 ( 1)  [xb]0b.sex + [xb]contactf_lag + [xb]4.isced_rec#c.contactf_lag = 0

------------------------------------------------------------------------------
             |     exp(b)   Std. err.      z    P>|z|     [95% conf. interval]
-------------+----------------------------------------------------------------
         (1) |   .9621875   .2291437    -0.16   0.871     .6033192    1.534519
------------------------------------------------------------------------------

.
. lincom c.contactf_lag + c.contactf_lag#1.isced_rec + 1.sex, eform

 ( 1)  [xb]1.sex + [xb]contactf_lag + [xb]1.isced_rec#c.contactf_lag = 0

------------------------------------------------------------------------------
             |     exp(b)   Std. err.      z    P>|z|     [95% conf. interval]
-------------+----------------------------------------------------------------
         (1) |   .4368338   .2438351    -1.48   0.138     .1462805    1.304506
------------------------------------------------------------------------------

. lincom c.contactf_lag + c.contactf_lag#2.isced_rec + 1.sex, eform

 ( 1)  [xb]1.sex + [xb]contactf_lag + [xb]2b.isced_rec#co.contactf_lag = 0
```

```
-----------------------------------------------------------------------------
             |     exp(b)   Std. err.      z    P>|z|     [95% conf. interval]
-------------+---------------------------------------------------------------
         (1) |   .4392315   .2402842    -1.50   0.133     .1503275     1.28336
-----------------------------------------------------------------------------

. lincom c.contactf_lag + c.contactf_lag#3.isced_rec + 1.sex, eform

 ( 1)  [xb]1.sex + [xb]contactf_lag + [xb]3.isced_rec#c.contactf_lag = 0

-----------------------------------------------------------------------------
             |     exp(b)   Std. err.      z    P>|z|     [95% conf. interval]
-------------+---------------------------------------------------------------
         (1) |   .5092875   .2767866    -1.24   0.214     .1755309    1.477653
-----------------------------------------------------------------------------

. lincom c.contactf_lag + c.contactf_lag#4.isced_rec + 1.sex, eform

 ( 1)  [xb]1.sex + [xb]contactf_lag + [xb]4.isced_rec#c.contactf_lag = 0

-----------------------------------------------------------------------------
             |     exp(b)   Std. err.      z    P>|z|     [95% conf. interval]
-------------+---------------------------------------------------------------
         (1) |   .3903746   .2293986    -1.60   0.109     .1233912    1.235034
```

Online Supplementary Material E

Cohort interactions

```
. tab cohort have_kids

                     |       have_kids
        Birth cohort |         0          1 |     Total
---------------------+----------------------+----------
         1 1991-1993 |     5,982        194 |     6,176
         2 1981-1983 |     7,153        611 |     7,764
         3 1971-1973 |     2,417         88 |     2,505
         4 2001-2003 |        97          1 |        98
```

```
----------------------+----------------------+----------
                Total |     15,649       894 |     16,543

. * only one birth from youngest cohort, so this one we drop

. drop if cohort == 4

(98 observations deleted)
```

Model

```
. stpm2 c.contactm_lag##b2.cohort c.contactf_lag##b2.cohort b2.isced_rec sex rela
german_native_nomigration parents_evernottogether siblings demodiff b2.cohort, scale(hazard) df(5)
tvc(rela

> ) dftvc(5) eform

note: delayed entry models are being fitted


Iteration 0:   log likelihood = -62.016487

Iteration 1:   log likelihood = -49.234617

Iteration 2:   log likelihood = -39.179454

Iteration 3:   log likelihood = -37.327884

Iteration 4:   log likelihood = -37.283079

Iteration 5:   log likelihood = -37.282997

Iteration 6:   log likelihood = -37.282997


Log likelihood = -37.282997                             Number of obs = 16,445


-------------------------------------------------------------------------------------------
                          |     exp(b)   Std. err.      z    P>|z|     [95% conf. interval]
--------------------------+----------------------------------------------------------------
xb                        |
             contactm_lag |   .9891474   .0415555    -0.26   0.795     .9109632    1.074042
                          |
                   cohort |
             1 1991-1993  |   .6331002   .3402246    -0.85   0.395     .2208216    1.815112
             3 1971-1973  |    .622153    .322141    -0.92   0.359     .2255063    1.716469
                          |
    cohort#c.contactm_lag |
             1 1991-1993  |   1.270902   .1183108     2.58   0.010     1.058941    1.525289
             3 1971-1973  |   .9283449   .1034711    -0.67   0.505     .7461678    1.155001
                          |
             contactf_lag |   1.108471   .0390846     2.92   0.003     1.034453    1.187784
```

```
                          |
     cohort#c.contactf_lag |
              1 1991-1993  |   .8427227    .050669    -2.85   0.004     .7490416    .9481203
              2 1981-1983  |          1   4.08e-17    -2.47   0.014            1           1
              3 1971-1973  |   1.120964   .1137415     1.13   0.260     .9188033    1.367606
                          |
                isced_rec |
                        1  |   1.761362   .2896302     3.44   0.001     1.276089    2.431175
                        3  |   .9395883   .0666474    -0.88   0.380     .8176355    1.079731
                        4  |   .4836577   .1000411    -3.51   0.000      .322458    .7254425
                          |
                      sex |   1.142639   .0783545     1.94   0.052     .9989399    1.307009
                     rela |   8.201399   2.454502     7.03   0.000     4.561857    14.74464
  german_native_nomigration |   .9622461   .0907545    -0.41   0.683     .7998432    1.157624
   parents_evernottogether |   1.074027   .0878712     0.87   0.383     .9149018    1.260828
                 siblings |   1.316493    .150893     2.40   0.016     1.051614    1.648091
                 demodiff |   1.181813   .1236985     1.60   0.110     .9626195    1.450917
                    _rcs1 |   1.777307   .3091734     3.31   0.001     1.263836    2.499391
                    _rcs2 |   1.079843   .1027276     0.81   0.419     .8961574    1.301179
                    _rcs3 |   1.026971   .0404779     0.68   0.500     .9506229    1.109451
                    _rcs4 |   1.002041   .0348534     0.06   0.953     .9360064    1.072735
                    _rcs5 |   1.010148   .0253055     0.40   0.687     .9617477    1.060983
               _rcs_rela1 |   1.890627   .3455715     3.48   0.000     1.321362    2.705139
               _rcs_rela2 |   1.195159   .1317181     1.62   0.106     .9629753    1.483325
               _rcs_rela3 |   1.102296   .0541454     1.98   0.047     1.001121    1.213695
               _rcs_rela4 |    1.04997   .0388433     1.32   0.187     .9765329    1.128929
               _rcs_rela5 |   .9890558   .0259887    -0.42   0.675     .9394082    1.041327
                    _cons |   .0225076   .0091664    -9.32   0.000     .0101314    .0500021
------------------------------------------------------------------------------------------
Note: Estimates are transformed only in the first equation.
```

Linear combinations

```
. lincom c.contactm_lag + c.contactm_lag#1.cohort, eform

 ( 1)  [xb]contactm_lag + [xb]1.cohort#c.contactm_lag = 0

------------------------------------------------------------------------------
             |     exp(b)   Std. err.      z    P>|z|     [95% conf. interval]
```

```
-------------+----------------------------------------------------------------
         (1) |   1.257109   .1050488     2.74   0.006     1.067194    1.480821
------------------------------------------------------------------------------

. lincom c.contactm_lag + c.contactm_lag#2.cohort, eform

 ( 1)  [xb]contactm_lag + [xb]2b.cohort#co.contactm_lag = 0

------------------------------------------------------------------------------
             |     exp(b)   Std. err.      z    P>|z|     [95% conf. interval]
-------------+----------------------------------------------------------------
         (1) |   .9891474   .0415555    -0.26   0.795     .9109632    1.074042
------------------------------------------------------------------------------

. lincom c.contactm_lag + c.contactm_lag#3.cohort, eform

 ( 1)  [xb]contactm_lag + [xb]3.cohort#c.contactm_lag = 0

------------------------------------------------------------------------------
             |     exp(b)   Std. err.      z    P>|z|     [95% conf. interval]
-------------+----------------------------------------------------------------
         (1) |   .9182699   .0947993    -0.83   0.409     .7500582    1.124206
------------------------------------------------------------------------------

. * cohort 1, i.e., 1991-1993 have children earlier when more contact with mother
.
. lincom c.contactf_lag + c.contactf_lag#1.cohort, eform

 ( 1)  [xb]contactf_lag + [xb]1.cohort#c.contactf_lag = 0

------------------------------------------------------------------------------
             |     exp(b)   Std. err.      z    P>|z|     [95% conf. interval]
-------------+----------------------------------------------------------------
         (1) |   .9341333   .0484011    -1.32   0.189     .8439269    1.033982
------------------------------------------------------------------------------

. lincom c.contactf_lag + c.contactf_lag#2.cohort, eform

 ( 1)  [xb]contactf_lag + [xb]2b.cohort#co.contactf_lag = 0
```

```
------------------------------------------------------------------------------
             |     exp(b)   Std. err.      z    P>|z|     [95% conf. interval]
-------------+----------------------------------------------------------------
         (1) |   1.108471   .0390846     2.92   0.003     1.034453    1.187784
------------------------------------------------------------------------------

. lincom c.contactf_lag + c.contactf_lag#3.cohort, eform

 ( 1)  [xb]contactf_lag + [xb]3.cohort#c.contactf_lag = 0

------------------------------------------------------------------------------
             |     exp(b)   Std. err.      z    P>|z|     [95% conf. interval]
-------------+----------------------------------------------------------------
         (1) |   1.242556   .1191914     2.26   0.024     1.029592    1.499571
------------------------------------------------------------------------------

. * cohort 2 and 3, i.e., 1981-1983 and 1971-1973 have children earlier when more contact with father
```

Online Supplementary Material F

Emotional closeness instead of contact

Model 3 (presented in Table 2)

```
. eststo isced3: stpm2 c.emo_m_lag##b2.isced_rec c.emo_f_lag##b2.isced_rec sex rela
german_native_nomigration parents_evernottogether siblings demodiff b2.cohort, scale(hazard) df(5)
tvc(re

> la) dftvc(5) eform

note: delayed entry models are being fitted


Iteration 0:   log likelihood = -56.706627

Iteration 1:   log likelihood = -46.681464

Iteration 2:   log likelihood = -38.945939

Iteration 3:   log likelihood = -37.530414

Iteration 4:   log likelihood = -37.517054

Iteration 5:   log likelihood = -37.517034

Iteration 6:   log likelihood = -37.517034


Log likelihood = -37.517034                             Number of obs = 16,132


------------------------------------------------------------------------------------------

                          |     exp(b)   Std. err.      z    P>|z|     [95% conf. interval]

--------------------------+---------------------------------------------------------------

xb                        |

                emo_m_lag |   1.137436    .068755     2.13   0.033     1.010356    1.280501

                          |

                isced_rec |

                       1  |   4.036648   2.700831     2.09   0.037      1.08768      14.981

                       3  |   1.852131   .6618045     1.72   0.085     .9194325    3.730985

                       4  |   .4561067   .5291041    -0.68   0.499     .0469499    4.430965

                          |

    isced_rec#c.emo_m_lag |

                       1  |   .9242843   .1488394    -0.49   0.625     .6741163    1.267291

                       2  |          1   1.58e-16   -0.86   0.391            1           1

                       3  |   .7755246   .0700237    -2.82   0.005     .6497388    .9256618

                       4  |   1.127752   .3256023     0.42   0.677     .6404073    1.985961

                          |

                emo_f_lag |   1.029555   .0510653     0.59   0.557       .93418    1.134668

                          |

    isced_rec#c.emo_f_lag |
```

```
                       1  |   .8581377   .1155567    -1.14   0.256     .6590742    1.117325
                       3  |   1.102337   .0853929     1.26   0.208     .9470564    1.283078
                       4  |   .8729055   .1863505    -0.64   0.524     .5744472     1.32643
                          |
                      sex |   1.155246   .0800782     2.08   0.037      1.00849    1.323358
                     rela |   9.808985   2.721955     8.23   0.000      5.69402    16.89776
german_native_nomigration |   .9540432   .0906004    -0.50   0.620     .7920161    1.149217
  parents_evernottogether |   .9926156   .0790795    -0.09   0.926     .8491173    1.160365
                 siblings |   1.288906   .1488828     2.20   0.028     1.027776    1.616384
                 demodiff |   1.139989   .1216626     1.23   0.220     .9248219    1.405216
                          |
                   cohort |
             1 1991-1993  |   1.097788   .1361596     0.75   0.452     .8608811    1.399889
             2 1981-1983  |          1   3.32e-16    -2.14   0.033            1           1
             3 1971-1973  |   .7068473   .1250069    -1.96   0.050     .4997922    .9996817
             4 2001-2003  |    8.95816   9.462863     2.08   0.038     1.129953    71.01941
                          |
                    _rcs1 |   1.958878   .3615497     3.64   0.000     1.364273    2.812636
                    _rcs2 |   1.112189   .1274759     0.93   0.354     .8884158    1.392326
                    _rcs3 |   1.035902   .0513605     0.71   0.477     .9399741     1.14162
                    _rcs4 |   .9966213   .0398482    -0.08   0.933     .9215022    1.077864
                    _rcs5 |   1.012693   .0280884     0.45   0.649     .9591106    1.069269
               _rcs_rela1 |   1.729084   .3316729     2.85   0.004     1.187241     2.51822
               _rcs_rela2 |   1.142408   .1459299     1.04   0.297     .8893846    1.467414
               _rcs_rela3 |   1.095228   .0635042     1.57   0.117     .9775735    1.227041
               _rcs_rela4 |    1.05782   .0444221     1.34   0.181     .9742409    1.148569
               _rcs_rela5 |    .986752   .0284695    -0.46   0.644     .9325012    1.044159
                    _cons |   .0164053   .0067447   -10.00   0.000     .0073287    .0367232
------------------------------------------------------------------------------------------
Note: Estimates are transformed only in the first equation.
```

Linear combinations (presented in Table 3)

```
. lincom c.emo_m_lag + c.emo_m_lag#1.isced_rec, eform


 ( 1)  [xb]emo_m_lag + [xb]1.isced_rec#c.emo_m_lag = 0


------------------------------------------------------------------------------
             |     exp(b)   Std. err.      z    P>|z|     [95% conf. interval]
```

```
-------------+----------------------------------------------------------------
         (1) |   1.051315   .1573056     0.33   0.738     .7840966      1.4096
------------------------------------------------------------------------------

. lincom c.emo_m_lag + c.emo_m_lag#2.isced_rec, eform

 ( 1)  [xb]emo_m_lag + [xb]2b.isced_rec#co.emo_m_lag = 0

------------------------------------------------------------------------------
             |     exp(b)   Std. err.      z    P>|z|     [95% conf. interval]
-------------+----------------------------------------------------------------
         (1) |   1.137436    .068755     2.13   0.033     1.010356    1.280501
------------------------------------------------------------------------------

. lincom c.emo_m_lag + c.emo_m_lag#3.isced_rec, eform

 ( 1)  [xb]emo_m_lag + [xb]3.isced_rec#c.emo_m_lag = 0

------------------------------------------------------------------------------
             |     exp(b)   Std. err.      z    P>|z|     [95% conf. interval]
-------------+----------------------------------------------------------------
         (1) |   .8821099   .0598021    -1.85   0.064     .7723533    1.007464
------------------------------------------------------------------------------

. lincom c.emo_m_lag + c.emo_m_lag#4.isced_rec, eform

 ( 1)  [xb]emo_m_lag + [xb]4.isced_rec#c.emo_m_lag = 0

------------------------------------------------------------------------------
             |     exp(b)   Std. err.      z    P>|z|     [95% conf. interval]
-------------+----------------------------------------------------------------
         (1) |   1.282746   .3624959     0.88   0.378      .737219    2.231952
------------------------------------------------------------------------------

.
. lincom c.emo_f_lag + c.emo_f_lag#1.isced_rec, eform

 ( 1)  [xb]emo_f_lag + [xb]1.isced_rec#c.emo_f_lag = 0
```

```
------------------------------------------------------------------------------
             |     exp(b)   Std. err.      z    P>|z|     [95% conf. interval]
-------------+----------------------------------------------------------------
         (1) |   .8835001   .1116583    -0.98   0.327     .6896525    1.131835
------------------------------------------------------------------------------

. lincom c.emo_f_lag + c.emo_f_lag#2.isced_rec, eform

 ( 1)  [xb]emo_f_lag + [xb]2b.isced_rec#co.emo_f_lag = 0

------------------------------------------------------------------------------
             |     exp(b)   Std. err.      z    P>|z|     [95% conf. interval]
-------------+----------------------------------------------------------------
         (1) |   1.029555   .0510653     0.59   0.557       .93418    1.134668
------------------------------------------------------------------------------

. lincom c.emo_f_lag + c.emo_f_lag#3.isced_rec, eform

 ( 1)  [xb]emo_f_lag + [xb]3.isced_rec#c.emo_f_lag = 0

------------------------------------------------------------------------------
             |     exp(b)   Std. err.      z    P>|z|     [95% conf. interval]
-------------+----------------------------------------------------------------
         (1) |   1.134917   .0696317     2.06   0.039     1.006328    1.279937
------------------------------------------------------------------------------

. lincom c.emo_f_lag + c.emo_f_lag#4.isced_rec, eform

 ( 1)  [xb]emo_f_lag + [xb]4.isced_rec#c.emo_f_lag = 0

------------------------------------------------------------------------------
             |     exp(b)   Std. err.      z    P>|z|     [95% conf. interval]
-------------+----------------------------------------------------------------
         (1) |   .8987044   .1874886    -0.51   0.609      .597088    1.352681
```

Model 4 (presented in Table 4)

```
. eststo isced4: stpm2 c.emo_m_lag##b2.isced_rec##sex c.emo_f_lag##b2.isced_rec##sex rela
german_native_nomigration parents_evernottogether siblings demodiff b2.cohort, scale(hazard) df(5)

> tvc(rela) dftvc(5) eform
```

```
note: delayed entry models are being fitted

Iteration 0:   log likelihood = -46.984171
Iteration 1:   log likelihood = -36.506794
Iteration 2:   log likelihood =  -28.84968
Iteration 3:   log likelihood = -27.469804
Iteration 4:   log likelihood = -27.457332
Iteration 5:   log likelihood = -27.457315
Iteration 6:   log likelihood = -27.457315

Log likelihood = -27.457315                        Number of obs = 16,132

------------------------------------------------------------------------------------------
                          |     exp(b)   Std. err.      z    P>|z|     [95% conf. interval]
--------------------------+---------------------------------------------------------------
xb                        |
               emo_m_lag  |   1.064769   .0965789     0.69   0.489     .8913489    1.271928
                          |
               isced_rec  |
                      1   |   3.230949   2.864065     1.32   0.186     .5685713    18.36011
                      3   |   1.049857    .524481     0.10   0.922     .3943592    2.794912
                      4   |     .30401   .4896618    -0.74   0.460     .0129383    7.143292
                          |
    isced_rec#c.emo_m_lag |
                      1   |   .8799194   .1941484    -0.58   0.562     .5709932    1.355985
                      2   |          1   5.14e-16     0.72   0.471            1           1
                      3   |   .7196172   .0996404    -2.38   0.017      .548582    .9439771
                      4   |   .9047807   .4194495    -0.22   0.829     .3646975    2.244677
                          |
                   1.sex  |   .6260756   .3175254    -0.92   0.356     .2316994    1.691721
                          |
          sex#c.emo_m_lag |
                      0   |          1   9.14e-17    -1.30   0.194            1           1
                      1   |   1.101307   .1342253     0.79   0.429     .8672923    1.398464
                          |
            isced_rec#sex |
                    1 0   |          1   1.51e-16     0.68   0.499            1           1
                    1 1   |   1.371319   1.880152     0.23   0.818     .0933459    20.14568
                    2 0   |          1   8.31e-17    -0.52   0.604            1           1
```

3 1	2.436455	1.759337	1.23	0.217	.5917186	10.03233
4 0	1	8.23e-17	1.11	0.265	1	1
4 1	1.988463	4.720983	0.29	0.772	.0189498	208.6553
isced_rec#sex#c.emo_m_lag						
1 0	1	3.00e-16	-2.27	0.023	1	1
1 1	1.167396	.3853401	0.47	0.639	.6112927	2.229396
2 0	1	1.57e-16	3.37	0.001	1	1
3 1	1.147802	.2124337	0.74	0.456	.7985972	1.649705
4 0	1	4.80e-17	-0.26	0.796	1	1
4 1	1.327097	.812322	0.46	0.644	.3998326	4.404808
emo_f_lag	.9760626	.0711532	-0.33	0.740	.8461097	1.125975
isced_rec#c.emo_f_lag						
1	.9773231	.1906881	-0.12	0.906	.6667423	1.432578
2	1	1.31e-16	1.84	0.066	1	1
3	1.456577	.1803557	3.04	0.002	1.142711	1.856652
4	1.242824	.5195461	0.52	0.603	.5477418	2.819961
sex#c.emo_f_lag						
1	1.109662	.1074668	1.07	0.283	.9178147	1.341611
isced_rec#sex#c.emo_f_lag						
1 0	1	8.44e-17	1.77	0.076	1	1
1 1	.7582295	.2055051	-1.02	0.307	.4457558	1.289746
2 0	1	2.47e-16	1.41	0.159	1	1
3 1	.6218786	.099146	-2.98	0.003	.4549868	.8499874
4 0	1	1.54e-16	-1.16	0.248	1	1
4 1	.6007228	.2922289	-1.05	0.295	.2315244	1.55866
rela	9.683758	2.664917	8.25	0.000	5.646742	16.60695
german_native_nomigration	.9420184	.0897012	-0.63	0.530	.7816385	1.135306
parents_evernottogether	1.006032	.0804958	0.08	0.940	.8600119	1.176845
siblings	1.281866	.1481909	2.15	0.032	1.021972	1.607853
demodiff	1.151776	.1231059	1.32	0.186	.9340899	1.420193
cohort						
1 1991-1993	1.078361	.1340378	0.61	0.544	.8452043	1.375836

```
         2 1981-1983  |          1   1.85e-17     0.19   0.853            1           1
         3 1971-1973  |   .6966674   .1232605    -2.04   0.041     .4925195    .9854339
         4 2001-2003  |   8.751453   9.281046     2.05   0.041     1.094884     69.9507
                      |
                _rcs1 |    1.95381    .356845     3.67   0.000     1.365898    2.794771
                _rcs2 |   1.115148    .127752     0.95   0.341     .8908786    1.395876
                _rcs3 |    1.03314   .0515882     0.65   0.514     .9368192    1.139364
                _rcs4 |   .9943713   .0396111    -0.14   0.887     .9196883    1.075119
                _rcs5 |   1.012543   .0278852     0.45   0.651     .9593382    1.068699
           _rcs_rela1 |   1.741794   .3308163     2.92   0.003      1.20041    2.527343
           _rcs_rela2 |    1.14331   .1460212     1.05   0.294     .8901231    1.468512
           _rcs_rela3 |   1.098546   .0641434     1.61   0.107     .9797538    1.231741
           _rcs_rela4 |     1.0596   .0443748     1.38   0.167     .9761008    1.150242
           _rcs_rela5 |   .9869439    .028299    -0.46   0.647     .9330086    1.043997
                _cons |   .0242237   .0115054    -7.83   0.000     .0095488    .0614514
------------------------------------------------------------------------------------------
Note: Estimates are transformed only in the first equation.
```

Linear combinations (presented in Table 5)

```
. lincom c.emo_f_lag + c.emo_f_lag#1.isced_rec + 0.sex, eform

 ( 1)  [xb]0b.sex + [xb]emo_f_lag + [xb]1.isced_rec#c.emo_f_lag = 0

------------------------------------------------------------------------------
             |     exp(b)   Std. err.      z    P>|z|     [95% conf. interval]
-------------+----------------------------------------------------------------
         (1) |   .9539286   .1735062    -0.26   0.795     .6678735    1.362503
------------------------------------------------------------------------------

. lincom c.emo_f_lag + c.emo_f_lag#2.isced_rec + 0.sex, eform

 ( 1)  [xb]0b.sex + [xb]emo_f_lag + [xb]2b.isced_rec#co.emo_f_lag = 0

------------------------------------------------------------------------------
             |     exp(b)   Std. err.      z    P>|z|     [95% conf. interval]
-------------+----------------------------------------------------------------
         (1) |   .9760626   .0711532    -0.33   0.740     .8461097    1.125975
```

```
------------------------------------------------------------------------------

. lincom c.emo_f_lag + c.emo_f_lag#3.isced_rec + 0.sex, eform

 ( 1)  [xb]0b.sex + [xb]emo_f_lag + [xb]3.isced_rec#c.emo_f_lag = 0

------------------------------------------------------------------------------
             |     exp(b)   Std. err.      z    P>|z|     [95% conf. interval]
-------------+----------------------------------------------------------------
         (1) |    1.42171   .1438293     3.48   0.001     1.165999    1.733501
------------------------------------------------------------------------------

. lincom c.emo_f_lag + c.emo_f_lag#4.isced_rec + 0.sex, eform

 ( 1)  [xb]0b.sex + [xb]emo_f_lag + [xb]4.isced_rec#c.emo_f_lag = 0

------------------------------------------------------------------------------
             |     exp(b)   Std. err.      z    P>|z|     [95% conf. interval]
-------------+----------------------------------------------------------------
         (1) |   1.213074   .4999257     0.47   0.639     .5408718    2.720696
------------------------------------------------------------------------------

.
. lincom c.emo_f_lag + c.emo_f_lag#1.isced_rec + 1.sex, eform

 ( 1)  [xb]1.sex + [xb]emo_f_lag + [xb]1.isced_rec#c.emo_f_lag = 0

------------------------------------------------------------------------------
             |     exp(b)   Std. err.      z    P>|z|     [95% conf. interval]
-------------+----------------------------------------------------------------
         (1) |   .5972314   .3213064    -0.96   0.338     .2080664    1.714286
------------------------------------------------------------------------------

. lincom c.emo_f_lag + c.emo_f_lag#2.isced_rec + 1.sex, eform

 ( 1)  [xb]1.sex + [xb]emo_f_lag + [xb]2b.isced_rec#co.emo_f_lag = 0

------------------------------------------------------------------------------
             |     exp(b)   Std. err.      z    P>|z|     [95% conf. interval]
```

```
-------------+----------------------------------------------------------------
         (1) |    .611089   .3216936    -0.94   0.349     .2177756    1.714746
------------------------------------------------------------------------------

. lincom c.emo_f_lag + c.emo_f_lag#3.isced_rec + 1.sex, eform

 ( 1)  [xb]1.sex + [xb]emo_f_lag + [xb]3.isced_rec#c.emo_f_lag = 0

------------------------------------------------------------------------------
             |     exp(b)   Std. err.      z    P>|z|     [95% conf. interval]
-------------+----------------------------------------------------------------
         (1) |   .8900982   .4599256    -0.23   0.822     .3233037    2.450559
------------------------------------------------------------------------------

. lincom c.emo_f_lag + c.emo_f_lag#4.isced_rec + 1.sex, eform

 ( 1)  [xb]1.sex + [xb]emo_f_lag + [xb]4.isced_rec#c.emo_f_lag = 0

------------------------------------------------------------------------------
             |     exp(b)   Std. err.      z    P>|z|     [95% conf. interval]
-------------+----------------------------------------------------------------
         (1) |   .7594758   .4959374    -0.42   0.674     .2111931    2.731167
------------------------------------------------------------------------------
```
